# NaroNet: Discovery of tumor microenvironment elements from highly multiplexed images

**DOI:** 10.1016/j.media.2022.102384

**Published:** 2022-02-14

**Authors:** Daniel Jiménez-Sánchez, Mikel Ariz, Hang Chang, Xavier Matias-Guiu, Carlos E. de Andrea, Carlos Ortiz-de-Solórzano

**Affiliations:** a Solid Tumors and Biomarkers Program, IDISNA, and Ciberonc, Center for Applied Medical Research, University of Navarra, Pamplona, 31008, Spain; b Biological Systems and Engineering Division, Lawrence Berkeley National Laboratory, CA, 94720, Berkeley, USA; c Department of Pathology, Hospital U Arnau de Vilanova and Hospital U de Bellvitge, Universities of Lleida and Barcelona, IDIBELL, IRBLLEIDA, CIBERONC, Lleida, 25198, Spain; d Department of Pathology, IDISNA, Ciberonc, Clínica Universidad de Navarra, University of Navarra, Pamplona, 31008, Spain

**Keywords:** Tumor microenvironment, Weakly supervised learning, Deep learning, Spatial biology, Multiplex imaging, Imaging mass cytometry, Cellular neighborhoods, Interpretable machine learning, Self supervised learning

## Abstract

Understanding the spatial interactions between the elements of the tumor microenvironment -i.e. tumor cells. fibroblasts, immune cells- and how these interactions relate to the diagnosis or prognosis of a tumor is one of the goals of computational pathology. We present NaroNet, a deep learning framework that models the multi-scale tumor microenvironment from multiplex-stained cancer tissue images and provides patient-level interpretable predictions using a seamless end-to-end learning pipeline. Trained only with multiplex-stained tissue images and their corresponding patient-level clinical labels, NaroNet unsupervisedly learns which cell phenotypes, cell neighborhoods, and neighborhood interactions have the highest influence to predict the correct label. To this end, NaroNet incorporates several novel and state-of-the-art deep learning techniques, such as patch-level contrastive learning, multi-level graph embeddings, a novel max-sum pooling operation, or a metric that quantifies the relevance that each microenvironment element has in the individual predictions. We validate NaroNet using synthetic data simulating multiplex-immunostained images where a patient label is artificially associated to the -adjustable-probabilistic incidence of different microenvironment elements. We then apply our model to two sets of images of human cancer tissues: 336 seven-color multiplex-immunostained images from 12 high-grade endometrial cancer patients; and 382 35-plex mass cytometry images from 215 breast cancer patients. In both synthetic and real datasets, NaroNet provides outstanding predictions of relevant clinical information while associating those predictions to the presence of specific microenvironment elements.

## 1. Introduction

The histopathology and phenotype of a tumor guide its diagnosis, prognosis, and help to predict its response to conventional or immune-based anticancer treatments. Indeed, cancers are graded based on tumor architecture and cellular morphology (histopathology), while the expression of relevant cancer biomarkers (phenotype) is used to stratify patients, predict their prognosis and customize their treatment. Automating these tasks using machine learning (ML) is the goal of a novel field known as computational pathology.

### 1.1. Computational pathology

Two main computational pathology strategies exist to automate the analysis of the histopathology of a tumor or its phenotype: weakly supervised deep learning (WSDL) and single cell analysis (SCA). WSDL builds on the widespread availability of whole slide imaging (WSI) to blindly extract prominent histopathological tumor features from large amounts of raw or weakly annotated images of H&E stained tissue sections. Trained only with patient-level labels, WSDL automatically associates these architectural tumor features with clinical labels (Veta et al., 2019; Srinidhi et al., 2021; van der Laak et al., 2021), eliminating the need for manual and extensive pixel-level annotations (Bulten et al., 2020). WSDL uses patches containing several cells as the basic unit of interpretability, to saliently localize tumor-specific regions. This strategy has been shown very effective, often outperforming human experts’ predictive ability. For instance, WSDL has been effectively used for tumor subtyping, patient grade classification, or lymph node metastasis detection without pathologist’s intervention (Campanella et al., 2019; Boehm et al., 2021; Bilal et al., 2021; Lu et al., 2021; Pinckaers et al., 2020; Cheng et al., 2021; Diao et al., 2021; Longo et al., 2021).

SCA emerged in the context of the research for novel cancer biomarkers, i.e., specific proteins that are expressed by tumor cells, defining their particular phenotype. This laborious task requires selecting potential targets from *in silico* data and validating these targets *in situ* to confirm that they are reliably related to a specific biological effect. Traditionally, this has been done one or a few markers at a time. Recently, the development of highly multiplexed tissue imaging technologies, such as imaging mass cytometry (IMC) or multiplex immunofluorescence (MI), allows simultaneous staining of tissue sections with a high number (>20) of biomarkers (Hao et al., 2021; Stopsack et al., 2020). These complex biomarker *signatures* provide a comprehensive visualization of the tumor microenvironment and the spatial relationship between its elements, which could be related to the biology and prognosis of the tumor (Rendeiro et al., 2021; Ji et al., 2020). However, the complexity of the patterns of expression and the spatial relationships between multiple markers exceeds the capabilities of the human brain. SCA methods (Schapiro et al., 2017) approach this task by first segmenting the cells in the tissue and quantifying their morphology and intensity of marker expression. This information is then used to find clusters of cells with similar phenotypes, as well as higher-order interactions or ‘neighborhoods’ between phenotypes (Schürch et al., 2020). To this end, SCA methods build topological networks containing cell phenotype interactions, and apply graph-based clustering (Blondel et al., 2008) to assign groups of cells to different neighborhoods. Since SCA methods use the cell as the basic unit of tissue representation, they provide a high level of interpretability. However, SCA methods are sequential and not learning-based, meaning that the phenotypes and neighborhoods extracted are inferred ignoring which were the clinical questions at hand, and therefore the quantified microenvironmental features are not necessarily optimal to differentiate patient types (McQuin et al., 2018). This is especially true when analyzing highly heterogeneous data, affected by technical non-linear variabilities caused by autofluorescence and/or low expression of some antigens (Jackson et al., 2020; Schürch et al., 2020).

### 1.2. Aim of the study

**Fig. 1.**
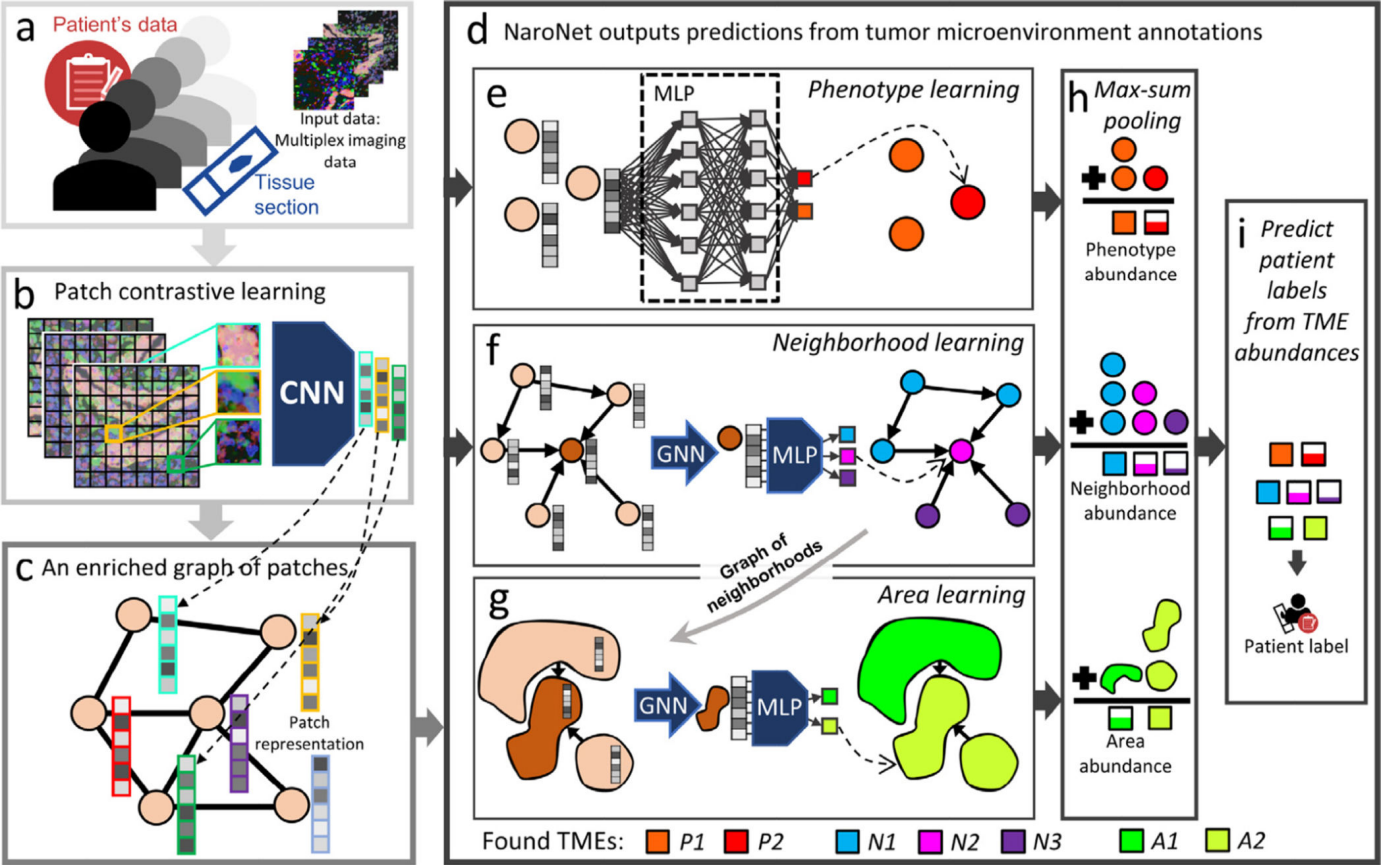
Scheme of NaroNet’s learning and discovery protocol. a. The input data consists of multiplex cancer tissue images with associated clinical and pathological information. b. The patch contrastive learning module divides images into patches and embeds each patch in a 256-dimensional vector using a CNN unsupervisedly trained to assign similar vectors to patches containing similar biological structures. c. An enriched graph of patches is generated that contains the spatial interactions between tissue patches. d. The graph of patches is fed to NaroNet: an interpretable ensemble of neural networks that learns phenotypes (e), phenotype neighborhoods (f), and areas of interaction between neighborhoods (g) to classify patients (i) based on the abundance of those tumor microenvironment elements (h). Legend. CNN: convolutional neural network; MLP: multilayer perceptron; GNN: graph neural network.

The aim of this study is to combine SCA (cell-level interpretable quantification of the tumor microenvironment) and WSDL (patch-based end-to-end learning of tumor histopathology) to automate the *in situ* discovery of tumor microenvironment elements (TMEs) that are relevant for a specific clinical predictive task. To this end, we have developed NaroNet, a multilevel, interpretable deep learning ensemble, which learns the most relevant TMEs from multiplex immunostained tissue sections while performing a classification task, using only patient-level labels. NaroNet assigns patches to TMEs at three levels of spatial complexity: local cell phenotypes, cellular neighborhoods, and interactions between neighborhoods that we name as tissue areas. The concept and main elements of NaroNet are illustrated in Fig. 1.

To validate NaroNet in a controlled way, i.e. having a ground truth, we first analyzed synthetic sets of multiplex images that simulate situations that can be found in real samples. Then we applied our model to learn relevant TMEs while predicting clinically relevant parameters from two real datasets: 336 7-plex images from 12 patients with high-grade endometrial cancer and a publicly available imaging mass cytometry dataset (Jackson et al., 2020) consisting of images from 215 breast cancer patients.

### 1.3. Methodological contributions

Besides the main conceptual novelty of discovering TMEs while performing clinical predictions from multiplex immunostained cancer tissues, NaroNet integrates novel and state-of-the-art ML approaches. In particular, the main methodological contributions of NaroNet are:

- The development of patch contrastive learning (PCL), a self-supervised learning algorithm that encodes high-dimensional pixel information into enriched patch-embeddings.
- The modelling of the tumor microenvironment in three levels of increasing complexity: local phenotypes, cellular neighborhoods and areas of interaction between cellular neighborhoods.
- A novel max-sum pooling operation that transforms TME learned annotations into patient-level vectors where each value specifies the incidence of TMEs.
- Two novel regularization loss terms that prevent NaroNet from producing spurious local minima: patch entropy loss and patient entropy loss.
- The optimal selection between several dataset-dependent architectural variations (e.g. multiple TME assignment, leveraging patch relevance, global reasoning unit, etc.). This is computationally feasible thanks to the dimensionality reduction provided by our PCL module.
- A BioInsights interpretability module that automates the association between patient types and TMEs, based on a novel predictive influence ratio (PIR) metric that quantifies the relevance that TMEs have in individual predictions.

The structure of the rest of this paper is as follows: Section 2 describes the synthetic and real datasets used, and describes the proposed methodology. Section 3 contains the experiments used to test the performance of NaroNet and reports the results obtained. Section 4 provides in-depth analysis of the proposed methods. Finally, we discuss the results in Section 5, and end with our conclusions in Section 6.

## 2. Materials and methods

### 2.1. Datasets

#### A. Synthetic patient cohorts.

**Fig. 2.**
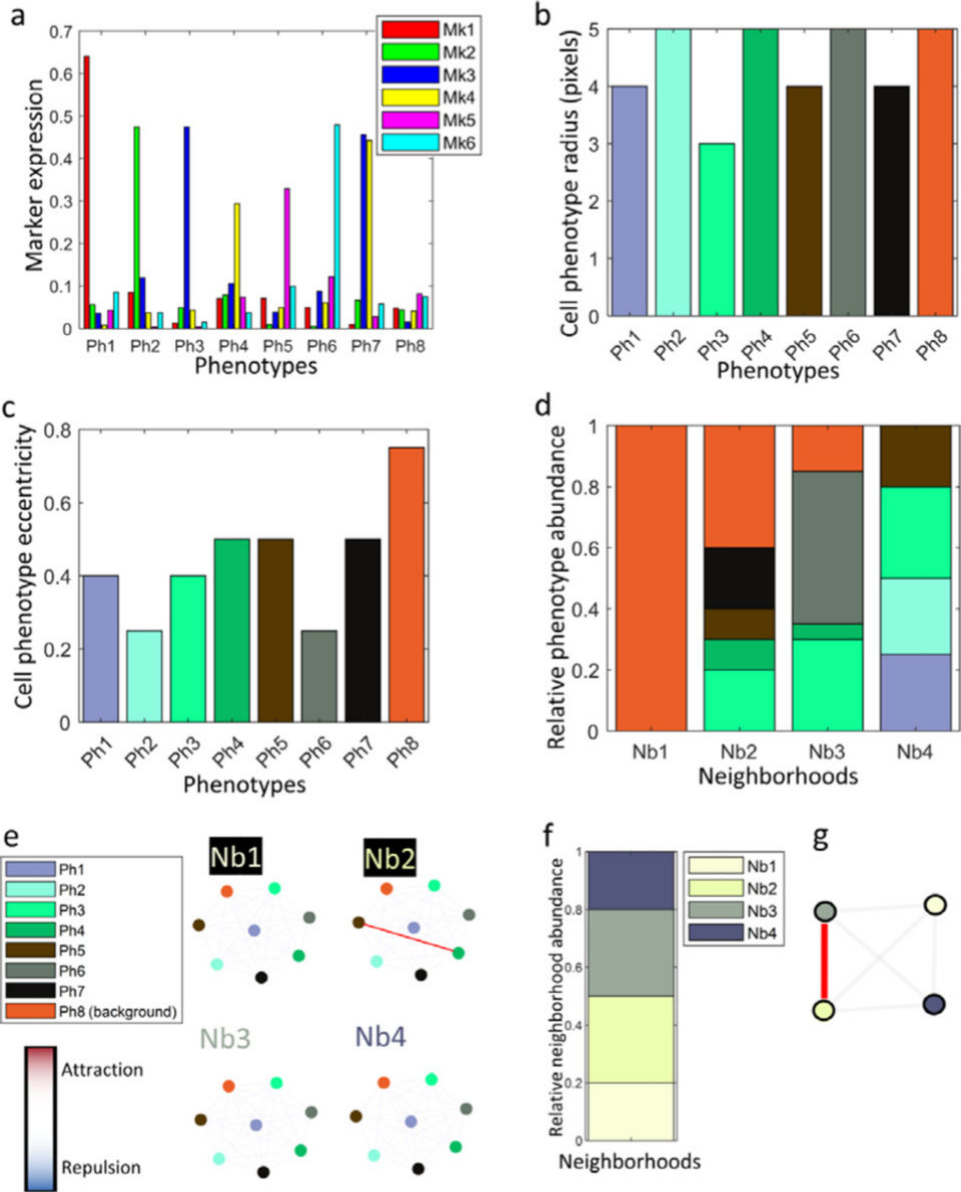
Synplex (synthetic simulator) default configuration. Eight cell phenotypes are defined by the expression of 6 markers (a), each of them having a specific cell size (b) and eccentricity (c). Four neighborhoods are defined based on the relative abundance of the phenotypes -please note that the color of neighborhood sections refer to the different phenotypes, as in pannels b and c-(d) and interaction, i.e. attraction/repulsion rules between phenotypes (e). These neighborhoods in turn are organized based on their frequency and interaction rules (f-g.).

An in-house developed multiplex immunostained tissue simulator (Jiménez-Sánchez et al., 2021), was used to create patient cohorts. Each patient of the cohort was represented by a 80 0×80 0 multiplex image that contained 8 cell phenotypes (Ph1-Ph8), defined by the (tunable) probabilistic level of expression of 6 fluorescently labeled markers (Mk1-Mk6) (Fig. 2a), the cell size (Fig. 2b), and shape (Fig. 2c). Four types of cell neighborhoods (Nb1-Nb4) were also defined based on the (adjustable) probabilistic abundance of the 8 cell phenotypes (Fig. 2d), and the (adjustable) interactions between them (Fig. 2e). Each neighborhood had a predefined prevalence in the tissue (Fig. 2f) and could interact with other neighborhoods (Fig. 2g) defining one area of interaction.

We simulated 7 patient cohorts. Each cohort contained 240 patients, distributed in 3 groups (type I, II, and III) of 80 patients each, defined by the variation of the default configuration parameters shown in (Fig. 2), simulating different disease paradigms in-spired on real scenarios:

##### Phenotype Marker Intensity (PMI).

In these patient cohorts, the cells of phenotype Ph6, located in neighborhood Nb3, displayed different relative intensity of Mk6 marker expression in each group of patients: 25% (type I), 50% (type II), or 75% (type III) (Supplementary Fig. 1). Two cohorts were created with different levels of complexity. In cohort PMI1, the relative abundance of Ph6 cells in Nb3 was set to 15% (moderately present), whereas in PMI2 the relative abundance of Ph6 was set to 0.25% (rarely present).

##### Phenotype Frequency (PF).

We simulated two patient cohorts where each group of patients displayed different abundance of cell phenotype Ph6. In PF1 (moderate presence) the relative abundance of Ph6 cells in neighborhood Nb3 was set to 0% (type I), 30% (type II), and 60% (type III) (Supplementary Fig. 2). In PF2 (rare presence), the relative abundance of Ph6 in Nb3 was set to 0% (type I), 0.12% (type II), and 0.25 (type III)%.

##### Cell-Cell Interactions (CCI).

We simulated two patient cohorts where cell phenotypes Ph4 and Ph5 that belong to neighborhood Nb2 repel (type I), show no interaction (type II), or attract (type III) (Supplementary Fig. 3). In cohort CCI1 (moderate presence) the relative abundance of both Ph4 and Ph5 in Nb2 was set to 5%; in CCI2 (rare presence), the relative abundance of both Ph4 and Ph5 was set to 1%.

##### Neighborhood-Neighborhood Interactions (NNI).

We simulated one patient cohort displaying different interactions between cellular neighborhoods, related to patient type. In this cohort (NNI1), Nb2 and Nb3 repel (type I), show no interaction (type II), or attract (type III). The relative abundance of both Nb2 and Nb3 was set to 15% (Supplementary Fig. 4).

#### B. Endometrial carcinomas.

Tissue sections from twelve Formalin-fixed, paraffin-embedded (FFPE) high-grade endometrial carcinomas were stained with a seven-color multiplex panel targeting key elements of the immune environment: CD4 and CD8 T cell membrane receptors, the transcription factor FOXP3, the bona fide T cell activation marker CD137 (4–1BB), the programmed cell death-1 (PD-1), cytokeratin (CK), and DAPI (counterstaining). 336 1876×1404×7 pixel images were obtained from the 12 tissue sections, using a Vectra-Polaris Automated Quantitative Pathology Imaging System (Perkin Elmer Inc., Waltham, MA, USA). Clinicopathological patient-level information was available for these tumors (León-Castillo et al., 2020), including the microsatellite instability (MSI) subclass, genomic copy number variation, and POLE mutation variants. A detailed description of this dataset, including the staining protocol and relevant clinicopathological information is included in Supplementary Materials: Appendix A. High grade Endometrial carcinomas.

#### C. Breast cancer.

A publicly available image dataset (Jackson et al., 2020) was used, consisting in 381 images from the same number of IMC (CyTOF) stained tissue sections, obtained from 215 breast cancer tumor biopsies. Tissue sections were stained with a 35-plex antibody panel staining clinically established breast cancer targets like oestrogen receptor (ER), progesterone receptor (PR), and HER2, as well as relevant oncogenes, signalling, and epigenetic related proteins.

### 2.2. Methodology: Patch Contrastive Learning (PCL) (Fig. 1b)

The goal of the first step of our pipeline is to convert each high-dimensional multiplex image of the cohort into a more manage-able list of low-dimensional embedding vectors. To this end, each image is divided into patches-our basic units of representation of the local tissue microenvironment, or phenotype-, and each patch is converted by the PCL module -a properly trained CNN- into a low-dimensional vector that embeds both the morphological and spectral information of the patch.

**Fig. 3.**
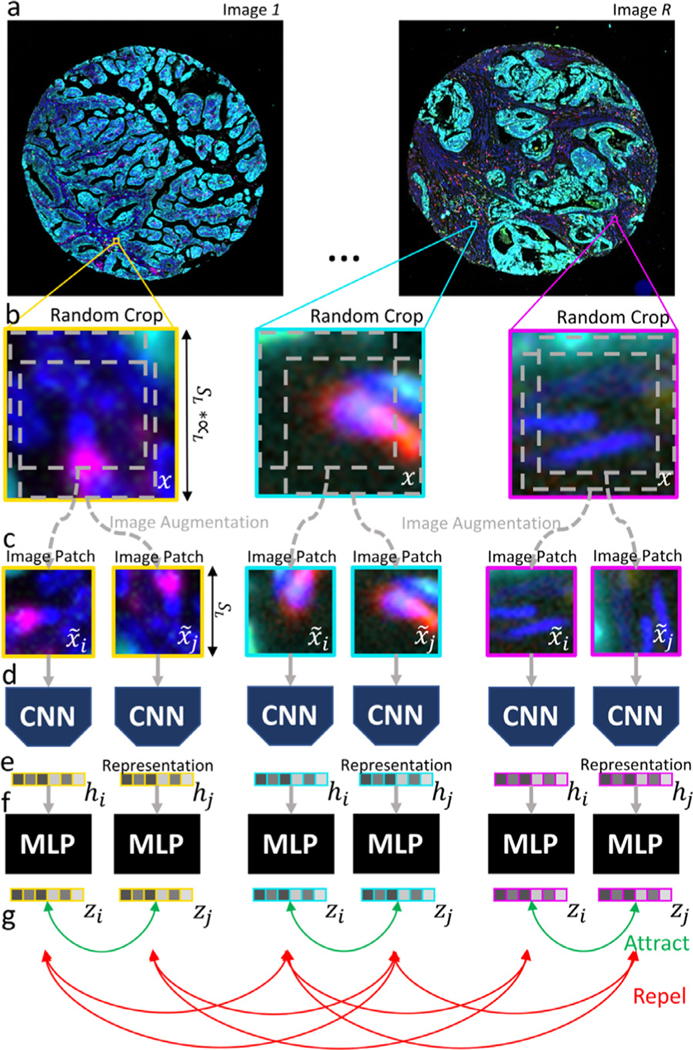
Visualization of Patch Contrastive Learning method description. Step-by-step illustration of Patch Contrastive Learning strategy.

The PCL module is trained iteratively. In each iteration, illustrated in Fig. 3, the PCL module is unsupervisedly trained to learn embeddings of a random set of patches, by maximizing the agreement between augmented views of highly overlapping patches, and minimizing the agreement between augmented views of distant patches, using a contrastive loss function. The choice of the image patch size *S_L_* is critical as it determines the extent to which biological structures can be captured, and their context. It also determines the size of the graph that is used to predict the outcome of the patient (see next section). This value was chosen considering that: *i.* a patch should be large enough to contain between zero and two cells, thus guaranteeing the interpretability of the model at the level of single cell or small cell environments; *ii.* the entire set of patches extracted from the images of one patient should fit in a single GPU, to efficiently generate patient predictions.

The steps required in each training iteration are described next:

#### A. Image crop selection.

A set ${\left\{x\right\}}_{1.B_{L}}$ is created made of *B_L_* image crops of size $\left(S_{L}*\propto_{L}\right)\times \left(S_{L}*\propto_{L}\right)\times B$ obtained at random positions of *R* random images chosen from the entire pool of *N* images of the cohort (Fig. 3a-b). Note that *B* is the number of spectral channels of the image.

#### B. Data augmentation.

An image patch set ${\left\{\tilde{x}\right\}}_{1.B_{L*2}}$ is created containing two augmented views, ${\tilde{x}}_{j1}$ and ${\tilde{x}}_{j2}$ each image crop $x_{j}$ of ${\left\{x\right\}}_{1.B_{L}}$. To this end, our data augmentation module (Fig. 3c) applies the following sequence of simple transformations to each image crop *X*_*j*_: *i*. of two *random crops* of size *S_L_*; *ii.* one *random rotation*; and *iii.* a *random cutout* consisting of masking out random 0.15 × *S_L_* sized sections of the patch.

#### C. Patch embedding generation.

The entire set of augmented patches ${\left\{\tilde{x}\right\}}_{1.B_{L*2}}$ is fed to a ResNet-101 (Fig. 3d), to obtain a set of *g* = 256 - dimensional vector representations or embeddings of the patches ${\left\{h\right\}}_{1\cdot B_{L*2}}$, being each patch, $h_{jk}=ResNet\left({\tilde{x}}_{jk}\right)$, k=1,2 where $h_{jk}\in {\mathbb{R}}^{g}$. Then, a multilayer perceptron (MLP) maps each representation $h_{jk}$ to a 128-dimensional vector $z_{jk}$.

#### D. Network parameter update.

Finally, a contrastive loss function is applied to ${\left\{z\right\}}_{1\cdot B_{L*2}}$ to create similar embeddings for patches contained in the same crop (i.e, $z_{j1}$ and $z_{j2}$) -possibly corresponding to the same biological structure-, while forcing dissimilar embeddings for patches contained in different image crops (i.e. $z_{ik}$ and $z_{ql}$, being i≠q)-possibly corresponding to dissimilar biological structures- (Fig. 3g). Let $sim(u,v)=u^{T}v/∥u∥∥v∥$ denote the cosine similarity between two vectors *u* and *v*. The loss function applied to any given pair of patches that belong to the same image crop is defined as:

(1) $$\ell_{j1,j2}=-\log\frac{exp\left(sim\left(z_{j1},z_{j2}\right)/\tau \right)}{\sum_{q=1,q\neq j,l=1,2}^{B_{L}-1}exp\left(sim\left(z_{jk},z_{ql}\right)/\tau \right)}$$

where *τ* is a temperature parameter set to 0.5.

This iterative (**A-D**) process is repeated until convergence to train the PCL module, which is next used to create vector embeddings of all the images of the cohort. To this end, each high dimensional multiplex image $i\in {\mathbb{R}}^{i_{x}\times i_{y}\times B}$ is divided in patches of size $S_{L}\times S_{L}\times B$, and each image patch is then introduced into the PCL module to obtain a patch embedding *h _j_* (Fig. 3e). This way the PCL module converts each image into a list of patch embeddings ${\left\{h\right\}}_{1.L}$. The resulting embedded image has reduced dimensionality, i.e. $i\in {\mathbb{R}}^{i_{x}\times i_{y}\times B}\to {\mathbb{R}}^{L\times g}$, where $L=\frac{i_{x}i_{y}}{S_{L}{}^{2}}$ is the number of patches of the image, and *g* is the number of features contained in the new patch embedding, in our case 256. This strategy reduces the image dataset size by approximately one order of magnitude.

### 2.3. Methodology: Patch-graph generation (Fig 1c)

A graph is then created that contains all the embedded patches of each tissue/image capturing cellular neighborhoods, i.e., local phenotypes that are spatially associated (Fig. 1c). This graph is 𝓖=(Z,A), where $Z\in {\mathbb{R}}^{Lxg}$ is a matrix that contains all the embeddings of the image ${\left\{h\right\}}_{1.L}$, and $A\in 0,1^{LxL}$ is an adjacency matrix that contains the connectivity between patches. To reduce the expensive memory burden of storing complete adjacency matrices, we ‘sparsify’ *A* as $A^{\prime }\in {\mathbb{Z}}^{Ex2}$, being $A^{\prime }$ a list of edges (i.e., connections) between patches, extracted from the non-zero values of the original *A*, where *E* is the number of edges present in the graph. Therefore, graph 𝓖=(Z,A), is converted into graph ${𝓖}^{\prime }=\left(Z,A^{\prime }\right)$. Since we connect each patch to its 4 adjacent neighbors, i.e., *E* = *L* × 4, the memory required to store *A*′ increases linearly with *L*, as opposed to *A*, which increases exponentially.

### 2.4. Methodology: NaroNet (Fig 1d)

Being $𝓓=\left(G_{1},y_{1}\right),\left(G_{2},y_{2}\right),\ldots ,\left(G_{M},y_{M}\right)$, a cohort of patients, where *M* is the number of patients, and each patient is represented by a graph $G_{m}\in 𝓖$, and a patient-level label $y_{m}\in 𝓨$, the goal of NaroNet is to learn a mapping $𝓖\overset{f}{\to }𝓨$ that relates patient information with patient labels, or predictions (Fig. 1d). The architecture of NaroNet is divided in two consecutive networks $𝓖\overset{f_{1}}{\to }(𝓟,𝓝,𝓐)\overset{f_{2}}{\to }𝓨$, trained end-to-end using the patient labels, where $𝓟\in {\mathbb{R}}^{P}$ is the abundance of local phenotypes (Fig. 1e), $𝓝\in {\mathbb{R}}^{N}$ is the abundance of neighborhoods (or phenotype interactions) (Fig. 1f), and $𝓐\in {\mathbb{R}}^{A}$ is the abundance of areas (or neighborhood interactions) (Fig. 1g). Note that *P*, *N*, and *A* are the number of phenotypes, neighborhoods or areas, respectively. We therefore model the tissue microenvironment using three levels of increasing spatial complexity. For the sake of consistency, we refer globally to 𝓟, 𝓝, 𝓐 as tumor microenvironment elements (TMEs). The first section of NaroNet, *f*_1_ (Fig. 1e-h), is an ensemble of three parallel networks that assigns nodes to distinct 𝓟, 𝓝, 𝓐 values. The second section, *f*_2_, assigns patient’s predictions from the learned TMEs (Fig. 1i). To learn the tumor microenvironment, the three neural networks $f_{1}=\left(f_{1P},f_{1N},f_{1A}\right)$ are trained in parallel from individual patient data and later pooled to obtain the abundance of each TME, as described in the following paragraphs:

#### A. Phenotype learning.

Each image patch, $h_{l}\in Z_{m}$, is assigned to a phenotype vector using $f_{1P}$, i.e.:

(2) $$S_{P}=f_{1P}\left(Z_{m}\right)\in {\mathbb{R}}^{L\times P}$$

where $f_{1P}$ is an 8-layer MLP with skip connections, with *P* phenotypes in the last layer. Therefore $f_{1P}$ takes the patch representations of image *Z_m_* and generates a patch assignment matrix *S_P_*, whose values represent the probability that each patch is assigned to *P* phenotypes.

#### B. Neighborhood learning.

Likewise, each image patch, $h_{l}\in Z_{m}$ is assigned to a neighborhood $f_{1N}$, i.e.:

(3) $$S_{N}=f_{1N}\left(Z,A^{\prime }\right)\in {\mathbb{R}}^{L\times N}$$

where $f_{1N}$ is a Graph Neural Network (GNN) followed by a 1-layer MLP which has an output dimensionality *N*. Therefore, $f_{1N}$ uses $Z_{m}$ patch representations and the adjacency matrix $A^{\prime }$ to produce a patch assignment matrix $S_{N}$. Here, the GNN captures relationships between connected patches of a graph. To that end, it performs *K* iterations of a trainable weighted sum of each graph node (in our case patch $h_{l}\in Z_{m}$) and its connected neighboring nodes, generating a new feature vector at the next hidden layer of the network (Hamilton et al., 2017; Kipf and Welling, 2017; Jimenez-Sanchez et al., 2020; Pati et al., 2020).

#### C. Area learning.

Each neighborhood that resulted from the previous GNN, $h_{l}\in {Z_{m}}^{\left(K\right)}$ is assigned to areas using a second $GNN\left(f_{1A}\right)$. To this end the following trainable assignment matrix is used:

(4) $$S_{I}=f_{1A}\left(S_{N}^{T}Z^{\left(K\right)},S_{N}^{T}A^{\prime S_{N}}\right)\in {\mathbb{R}}^{N\times A}$$

This GNN learns the higher order interactions between the *N* neighborhoods of the original graph. For this purpose, $f_{1A}$ is fed with the embeddings from *N* neighborhoods ${S_{N}}^{T}Z^{\left(K\right)}\in {\mathbb{R}}^{N\times H}$ and the interactions between neighborhoods ${S_{N}}^{T}A^{\prime S_{N}}\in {\mathbb{R}}^{N\times N}\cdot f_{1A}$ accumulates feature vectors of neighborhoods that are close to each other. As in the previous section, the GNN is applied *K* iterations or hops, this number indicating the extent to which the patch embeddings can capture information of their neighbors.

#### D. Max-sum pooling.

After applying $f_{1P},f_{1N},f_{1A}$, each row of *S_P_* contains the probability that each patch of the image contain each of the *P* phenotypes, each row of *S_N_* contains the probability that a patch of the image contain each of the *N* neighborhoods, and each row of *S_A_* contains the probability that a neighborhood of the image contain each of the possible *A* areas. The final step of *f*_1_ is a max-sum pooling operation that captures the abundance of each TME:

(5) $$𝓟=\sum_{1.L}\underset{1.P}{\max}\left(softmax\left(S_{P}\right)\right)\in {\mathbb{R}}^{P}$$

(6) $$𝓝=\sum_{1.L}\underset{1.N}{\max}\left(softmax\left(S_{N}\right)\right)\in {\mathbb{R}}^{N}$$

(7) $$𝓐=\sum_{1.L}\underset{1.A}{\max}\left(softmax\left(S_{A}\right)\right)\in {\mathbb{R}}^{A}$$

where $S_{P},S_{N},S_{A}$ (eqs. 5, 6, 7) are the assignment matrices whose values correspond to neuron activations, where the *softmax* activation function transforms them into probabilities in a row-wise fashion. The *max* operator function is applied row-wise so that only the maximum values of each row are kept, while the others are set to zero. The sum operator is applied column-wise to obtain the abundance of each TME. The resulting (𝓟, 𝓝, 𝓐) are the TME abundances that represent each patient.

The TME abundance vector $(𝓟,𝓝,𝓐)\in {\mathbb{R}}^{P+N+A}$ is fed to the second’s network section (*f*_2_), consisting in a 1 layer MLP, i.e., $y^{\prime }=f_{2}(𝓟,𝓝,𝓐)\in {\mathbb{R}}^{0}$, where $y^{\prime }$ is the prediction between *O* possible patient-outcomes. A cross entropy loss is used to train the parameters of both *f*_1_ and *f*_2_. The strategy used to implement *f*_1_ can produce spurious local minima where all patches are assigned to a single microenvironment element. This local optimal solution traps the gradient-based optimization, and reduces NaroNet’s performance. To prevent this, we use two regularization loss functions.

#### E. Patch entropy loss.

Patch entropy loss is used to regularize the probabilities given by eqs. 5, 6, 7. After initialization, the assignment of patches to TMEs is uncertain and the entropy of the patches is high. During the training process, we aim at knowing the assignment of patches to TMEs, obtaining a sparse matrix assignment. To this end, we propose to reduce patch entropy for each TME using a loss function:

(8) $$\ell =\frac{1}{L}*\sum_{l=1.L}-sum(softmax(S)*\log(softmax(S)))$$

where *S* is any of the matrices $S_{P},S_{N},S_{A}$, and the function generates $\left(\ell_{ep},\ell_{en},\ell_{ea}\right)$ losses, respectively. The final loss is restricted to ℝ∩[0,1] where the lower the value the most certain it is that a patch belongs to a specific TME. The final, combined loss is regularized by a *λ* parameter.

(9) $$\ell_{e}=\left(\lambda_{ep}*\ell_{ep}+\lambda_{en}*\ell_{en}+\lambda_{ei}*\ell_{ei}\right)/3$$

where $\lambda_{ep},\lambda_{en},\lambda_{ei}$ regularize how much the weights are adjusted to each (𝓟,𝓝,𝓐) TME. This is a specific learning rate that is chosen based on the tumor microenvironment complexity.

#### E. Patient entropy loss.

Patient entropy loss is used to avoid graph pooling collapse in (𝓟,𝓝,𝓐) TMEs.:

(10) $$\ell_{pp}=sum(𝓟*\log(𝓟)$$

(11) $$\ell_{pn}=sum(𝓝*\log(𝓝))$$

(12) $$\ell_{pa}=sum(𝓐*\log(𝓐))$$

where (𝓟,𝓝,𝓐) are the TME abundances and the vector $\left(\ell_{pp},\ell_{pn},\ell_{pa}\right)$ contains the calculated losses, their values being restricted to ℝ∩[−1,0]. As done for the patch entropy loss, the final loss is also regularized using a *λ* parameter, $\ell_{p}=lambda_{pp}*\ell_{pp}+\lambda_{pn}*\ell_{pn}+\lambda_{pa}*\ell_{pa})/3$. Notice that the lower the value of *l_p_* the most spread out is the abundance of the TMEs. This strategy is less restrictive than the orthogonal loss (Bianchi et al., 2020) since the regularization term does not force clusters to have the same size.

In order to provide the highest predictive and interpretability performance, NaroNet’s parameters and architecture variations (Supplementary Materials: Appendix C. Architectural variations) are optimally selected by an architecture search algorithm (Supplementary Materials: Appendix B. Architecture search).

### 2.5. Methodology: BioInsights interpretability module

Besides generating predictions, NaroNet identifies the elements of the tumor landscape that relate to a specific predictive task. This can be done *a posteriori* through the analysis of the TMEs (𝓟,𝓝,𝓐), obtained by NaroNet while classifying patients. That is the goal of the BioInsights module, that is done through the identification of global cohort-differentiating features (differential TME analysis), and relevant TMEs in individual predictions (predictive influence ratio).

#### A. Differential TME analysis.

NaroNet’s *f*_2_ network maps TME abundances to patient-outcomes, i.e., $𝓨=f_{2}{\left(𝓟,𝓝,𝓐\right)}_{1.M}$. Therefore, ${\left(𝓟,𝓝,𝓐\right)}_{1.M}$ are the coefficients or covariates of the model, and the patient’s predictions are made solely using the relative abundance of specific TMEs. We use regression analysis to interrogate which TMEs were more important to perform patient predictions. Specifically, to evaluate whether a specific TME is significant to perform patient predictions, a leave-one-out strategy is used, where a TME *t* is extracted from the set of all patient TME abundances ${\left(𝓟,𝓝,𝓐\right)}_{1.M}$ obtaining a new set of TMEs defined as ${\left(𝓟,𝓝,𝓐\right)}_{1.M}^{t}$. The model is evaluated with the entire patient cohort, and new prediction probabilities are obtained. Then, a Kruskal-wallis test is used to compare the prediction performance of the original TMEs with that of the leave-one-out model. If the null hypothesis is accepted, the extracted TME is considered to have predictive value.

#### B. Predictive influence ratio (PIR).

The differential TME analysis finds global patterns in patient cohorts but ignores the heterogeneity existing between patients/tissues. To address this, we introduce the predictive influence ratio (PIR), which quantifies the influence that each TME has on the prediction accuracy of a patient m∈1−M:

(13) $$PIR_{m,t}=\frac{f_{2}{\left(𝓟,𝓝,𝓐\right)}_{m}}{f_{2}{\left(𝓟,𝓝,𝓐\right)}_{m}^{t}}$$

where $PIR_{m,t}$ is the predictive influence ratio for a patient *m* and a TME *t*, and $f_{2}{\left(𝓟,𝓝,𝓘\right)}_{m}^{t}$ is the leave-one-out model performance. The higher the value of $PIR_{m,t}$ the most important the TME *t* is for the classification of patient *m*.

## 3. Results

### 3.1. Synthetic experiments

Seven patient cohorts, 240 patients each, were simulated (see Section 2.1.A), representing four disease paradigms (PM, PF, CCI and NN) in which either the moderate (1) or rare (2) presence of a specific TME differs between each of the three patient types (I, II or III). Seven different experiments were carried out, in which NaroNet was trained to predict the correct patient type for all patients of a cohort, while learning the TMEs that were relevant for that prediction. With these experiments we wanted to validate the ability of NaroNet to correctly classify each patient, and also to identify the relevant TME that defines each paradigm, in a properly controlled, quantifiable fashion (Supplementary Materials: Appendix D. Interpretability performance measure). In all 7 experiments, 120 patients of the cohort (40 patients of each type) were used for training and validation of NaroNet, including PCL and 500 runs of architecture search, and the remaining 120 patients were used for testing. Three train-test runs were made and averaged to report the final performance values.

The results obtained in each of these 7 experiments, in terms of NaroNet’s predictive accuracy, i.e. how accurately NaroNet predicted the patient type, and interpretability, i.e. the correspondence between the TME found more relevant by NaroNet and the TME that actually defined the disease paradigm, are shown in Table 1. Overall, the model predicts remarkably well all disease paradigms, even in those experiments involving rare cell populations.

#### Illustrative example: CCI1.

**Fig. 4.**
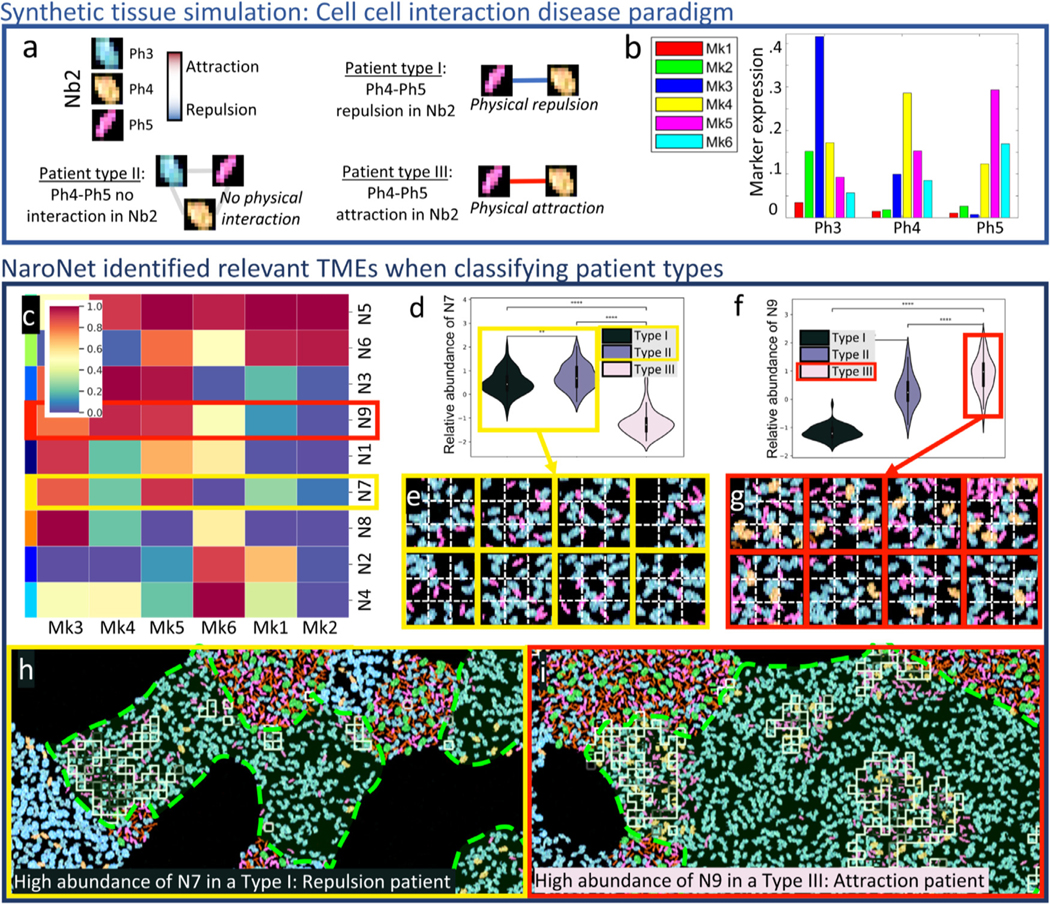
Graphical description of synthetic experiment CCI1. a. Ground truth: Schematic description of the interactions between cell phenotypes Ph3, Ph4, and Ph5 (located in neighborhood Nb2) that define each patient type (I-III). b. Marker expression levels for the three relevant cell phenotypes. c. Z-scored mean expression of all markers in the neighborhoods learned by NaroNet. d. Relative abundance of learned neighborhood N7 in the three patient groups. e. Representative patches assigned to N7. f. Relative abundance of learned neighborhood N9 in the three patient groups. e. Representative patches assigned to N9. h. Example of patient correctly classified as Type I (i.e. displaying Ph4-Ph5 repulsion), with squares showing patches assigned to learned neighborhood N7, located in ground truth neighborhood Nb2 (marked in red). i. Example of patient correctly classified as Type III (Ph4-Ph5 attraction), with squares showing patches assigned to learned neighborhood N9, located in ground truth neighborhood Nb2. (***p < 0.001; **** p < 0.0001).

Now we illustrate NaroNet’s methodology, results and interpretability using one of the synthetic experiments (CCI1) consisting of a patient cohort where cell phenotypes Ph4 and Ph5, coexisting in neighborhood Nb2, repel, show no interaction, or attract each other in patient types I, II, and III, respectively (Fig. 4a,b). The PCL module was trained to generate 256-long vector embeddings of 10×10 pixel patches with 52.8% contrastive accuracy, which was comparable to state-of-the-art semi-supervised learning setups (Chen et al., 2020b) (Table 1). Next, we used the training image set (120 patients) to calculate the optimal architecture (Supplementary Tables 1 and 2, Supplementary Figs. 5 and 6) and train the model. Then the test image set (120 patients) was used to calculate the classification performance. The receiver operating characteristic (ROC) curves, confusion matrix, and training and test accuracy curves obtained are shown in Supplementary Fig. 7. As shown in Table 1, the overall accuracy achieved for experiment CCI1 was 98.6% with a 95% confidence interval (CI) of [97.7,99.5]

Regarding the interpretability of the results, our global differential TME analysis revealed that, amongst all the neighborhoods detected by NaroNet (Supplementary Figs. 8,9), four neighborhoods -in order of statistical significance: N3, N7, N9, and N1-were most responsible for NaroNet’s predictions (Supplementary Fig. 10a). We also found that N3 and N7 are the most abundant neighborhoods in type I patients (repulsion between Ph4 and Ph5 cells)(Supplementary Figs. 10b-c), N9 is the most abundant neighborhood in type III patients (those displaying attraction of Ph4 and Ph5 cells)(Supplementary Figs. 10b-d), and N1 is the most abundant neighborhood in type II patients (no interaction between Ph4 and Ph5 cells) showing an equilibrium between attraction and repulsion (Supplementary Figs. 10e). The combination of these four neighborhoods overlaps 92.8% with ground truth neighborhood Nb2 (Table 1 and Fig. 4a). Therefore, NaroNet has correctly identified, and weighted in the classification, the tissue regions where the patient-defining TMEs are located.

We can next analyze the content of these four neighborhoods to confirm this finding: N7 contains high expression of markers Mk3 and Mk5, corresponding to cell phenotypes Ph3 and Ph5 (Fig. 4b,c). In type I patients, the abundance of N7 is statistically higher than in type III patients (Fig. 4d). If we look at N7 in type I tissues (Fig. 4e), we can confirm that it contains Ph3 and Ph5 cells, but not Ph4, meaning that there is physical repulsion between Ph4 and Ph5 as expected for this disease paradigm. The behaviour of N3 is similar to that of N7. N9 contains high expression of markers Mk3, Mk4 and Mk5, which correspond to cell phenotypes Ph3, Ph4, and Ph5 (Fig. 4b,c) and is significantly more abundant in type III patients, compared to patient types I and II (Fig. 4f). If we go back to the tissues of type III (Fig. 4g), it can be confirmed that N9 contains spatially related cells with phenotypes Ph4 and Ph5, as expected in this disease paradigm. Therefore, we have shown that the TMEs learned by NaroNet capture the specifics of the underlying disease paradigm and lead to a successful classification.

To interpret why an individual image/patient was classified as a certain patient type, we calculated the predictive influence ratio (PIR) value for each TME. This strategy, applied to CCI1 shows (Supplementary Fig. 11a) that for most type I patients, the abundance of neighborhood N7 was the most determinant classification factor. Conversely, N9 was highly predictive for type III patients, and N1 was highly relevant to successfully classify type II patients. We illustrate this with examples of individual predictions: a patient classified as type I with prediction confidence of 97.75% and a PIR value of 2.28 for N7 (Fig. 4h), and a patient classified as type III with prediction confidence of 94.36% and a PIR value of 1.61 for N9 (Fig. 4i).

### 3.2. Endometrial carcinomas

**Fig. 5.**
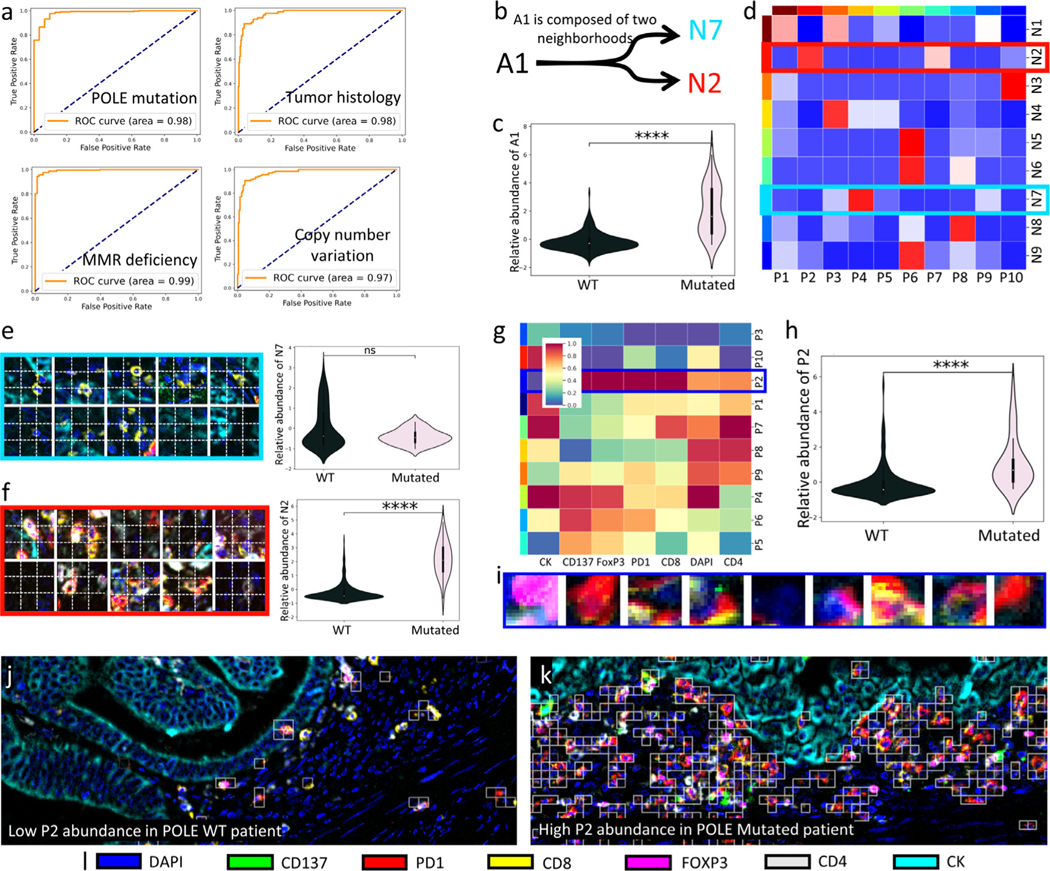
Association of high-grade endometrial carcinomas with patient-level labels. a. ROC curves showing the classification performance of NaroNet for the four tissue characteristics learned. b. Neighborhood composition of learned area A1. c. Violin-plot showing relative abundance of learned area A1 as a function of POLE mutation status. d. Heatmap showing interactions between the local phenotypes learned by NaroNet. e,f. Patches assigned to neighborhoods N7 and N2, and their corresponding abundance across patient-types. g. Heatmap showing the mean marker expression, for the phenotypes learned by NaroNet. h,i Patches assigned to phenotype P2 and its corresponding abundance across patient types. j,k. Images of WT and POLE mutated patients that were classified due to phenotype P2 abundance. White squares represent patches assigned to P2.

We first asked NaroNet to learn TMEs associated to four patient-level labels: the somatic POLE mutation, copy number variation (CNV), DNA mismatch repair (MMR) deficiency, and two tumor histology types (endometrial carcinoma or serous-like carcinoma) from 382 images of 12 high-grade endometrial carcinomas. The PCL module was trained to generate 256-dimensional embeddings of 15×15 pixel image patches, obtaining a high contrast accuracy of 81.11%. A 10-fold nested cross validation strategy was then used to optimize NaroNet’s parameters and hyperparameters. Using this strategy, the architecture search is repeated ten times (outer loop) using a 10-fold partition of the data. In each inner loop, 50 runs of the architecture search are implemented from 90% of the image dataset (344 images). The best architecture configuration (Supplementary Table 3) was then evaluated on the corresponding test fold of the outer loop (38 images), providing image-level predictions with average accuracy of 93.75% with 95% CI [91.16,96.33] (Fig. 5a and Supplementary Fig. 12) for the four patient-level labels.

As an example of the global interpretability of the results, we next analyze the interpretability of the model while predicting the POLE mutation status: NaroNet unsupervisedly learned 26 TMEs (Supplementary Figs. 13 and 14). Our differential TME composition analysis revealed that area A1 (p-value: 2.56×10^−9^) is the most predictive TME when making patient predictions. Particularly, A1 is significantly more associated to tumors harboring POLE mutations than to POLE wild type (WT) tumors (Fig. 5c). Area A1 contains neighborhoods N2 and N7 (Fig. 5b and Supplementary Fig. 14d) which in turn contain local phenotype interactions between P2-P7 and P4-P9, respectively (Fig. 5d). N7 contains CK+ tumor cells (P4) and intratumoral cells expressing CD4 and CD8 (P9), and by itself is not associated with POLE mutation (Fig. 5e). By contrast, N2 contains non-infiltrating cells that express CD8, PD1 and FoxP3 (P2) associated to tumor CK+ cells (P7), and is associated with POLE mutated patients (Fig. 5f). Furthermore, P2 by itself was significantly more abundant in POLE mutated patients (p-value: 2.80×10^−10^) compared to patients carrying the wild type version of the gene (Fig. 5g-i). All these findings are consistent with the literature as CD4, CD8, FoxP3, and PD1 are inflammation markers, and POLE-mutated endometrial carcinomas, usually with a better prognosis than POLE WT, with higher abundance of A1 areas, are described to have large lymphocyte populations (Li et al., 2019b). In summary, area A1 contains cellular neighborhoods related to high immunological activity, and points at the existence of interactions between specific immune phenotypes in POLE vs. non POLE mutated cancers that could be further explored, as could be done with other TMEs selected by NaroNet.

To illustrate the individual interpretability of our results we provide two examples of images in which phenotype P2 was the most relevant TME selected by NaroNet. The first image was correctly classified as POLE WT with a prediction confidence of 95.45%, and PIR value of 1.49. It shows a cold tumor landscape -low P2 abundance - that is associated with POLE WT patients (Fig. 5j). This is consistent with the global cohort-level findings (Fig. 5h). The second image was correctly classified as a POLE mutated with a prediction confidence of 99.24%, and a PIR value of 1.38. It shows a hot tumor landscape with high P2 abundance (Fig. 5k), being also consistent with global cohort-level findings.

#### Comparison with single cell analysis: Qupath.

To further validate NaroNet, we quantified the phenotype that NaroNet identified as the most discriminative between patient types (P2, i.e. high expression of CD8, PD1, and FoxP3), using QuPath (Bankhead et al., 2017), a widely used open source software for computational pathology (Supplementary Materials: Appendix E. Image analysis with Qupath). For each image of the cohort, we first quantified the level of expression of CD8, PD1, and FoxP3 from the cell segmentation masks obtained using the DAPI -counterstained-channel. Then we calculated the number of CD8+PD1+FoxP3+ cells and correlated this number with the number of patches that NaroNet assigned to P2, obtaining positive correlation (R^2^ = 0.63) (Supplementary Fig. 15b). Moreover, the respective violin-plots (Supplementary Fig. 15c-d) showed both QuPath and NaroNet are able to robustly distinguish patient-types based on CD8+PD1+FoxP3+ phenotype abundance, the outstanding difference being that NaroNet infers it without human supervision.

#### Patient-wise quantification.

Finally, to test NaroNet’s predictive power classifying subjects, i.e. patients and not individual images, based on the POLE mutation, we performed a leave-one-out experiment: iteratively, 11 patients (represented by all their images) were used to train the model and one patient was used for testing. Patient-wise predictions were calculated as the mean prediction value of all images that correspond to the test patient, achieving an overall accuracy of 83.33% with a 95% CI [63.02– 10 0.0 0%], and an AUC of 0.67 with a 95% CI [0.32–1].

### 3.3. Breast cancer cohort

**Fig. 6.**
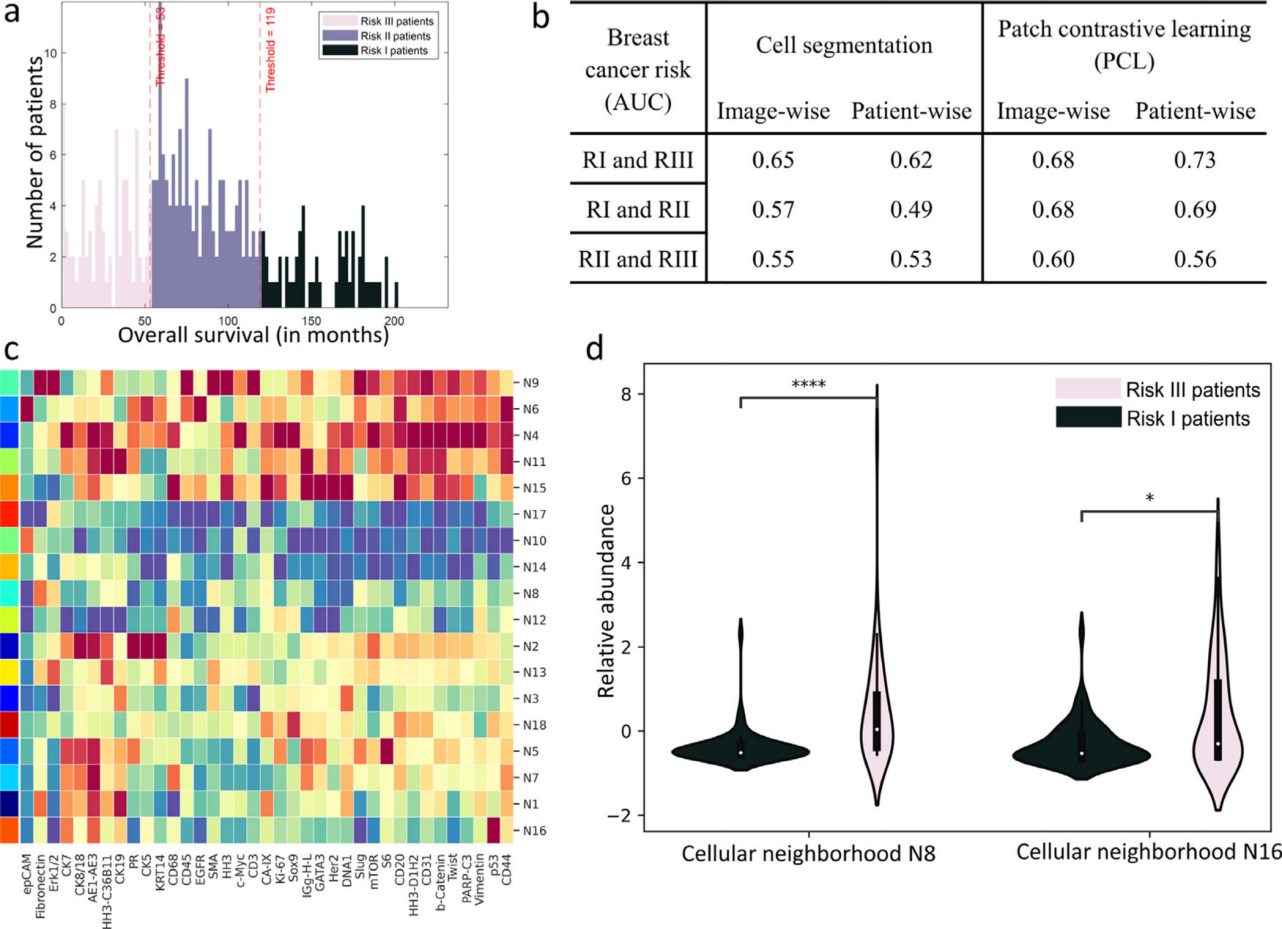
Association of spatially-resolved 35-plex of breast cancer tissues with patient long term survival. a. Histogram plot of patient overall survival in months, colored by risk classes. b. AUC prediction performance of NaroNet for four training strategies: image-wise or patient-wise, and using the cell segmentation provided with the image dataset or using the proposed PCL method. c. Heatmap showing the mean marker expression, for all neighborhoods learned by NaroNet. d. Violin-plot showing the relative abundance of learned neighborhoods N8 and N16 as a function of risk group, RI and RIII (p-values were adjusted with Bonferroni correction).

NaroNet was trained to associate TMEs with patient survival risk (Jackson et al., 2020). 215 patients were clustered by k-means into three risk groups (RI, RII, RIII) based on their long-term survival (Fig. 6a): RI contained 48 patients that survived more than 120 months, RII contained 107 patients that survived between 54 and 119 months, and RIII contained 60 patients that survived less than 53 months. The PCL module produced 18×18 pixel patch embeddings for all the images of the cohort, with a high contrast accuracy of 82.50%. As more than one image was acquired per patient, images from the same patient were combined in one single data structure (i.e., graph) and fed to NaroNet. A 10-fold nested cross validation was used to optimize NaroNet’s parameters and hyperparameters (Supplementary Table 4) as explained for the endometrial carcinoma experiment in Section 3.2. NaroNet predicted RI vs. RIII patients with an accuracy of 70.37% with 95% CI [61.81–78.92] and an AUC of 0.73 with 95% CI [63.15–82.00] (Supplementary Fig. 16).

As an example of how to make use of its global interpretability, NaroNet learned 57 distinct spatial patterns of TMEs able to predict the patient risk group (Fig. 6c and Supplementary Fig. 17). Using our differential TME composition analysis we found that a combination of two neighborhoods (N8 and N16) was significantly predictive (p-value < 0.05) when distinguishing between RI and RIII patients. N8 is a neighborhood that contains tumor cells (cytokeratin AE1/AE3 and cytokeratin 7 positive), and high presence of fibronectin. This neighborhood is more abundant in risk III patients (Fig. 6d). Fibronectin is a key component of the extracellular matrix. In particular, as seen in the literature, fibronectin is highly present in the remodeled tumor extracellular matrix, forming a barrier for the infiltration of immune cells. Consistent with our findings, fibronectin was associated with poorer patient survival (Fernandez-Garcia et al., 2014). Moreover, the abundance of neighborhood N16, consisting of tumor cells expressing p53, was also associated with bad prognosis (Fig. 6d). The tumor suppressor gene p53 is one of the most commonly mutated gene in human cancers. TP53 gene mutation is generally associated with a strong and diffuse immunoexpression of p53. Consistent with our findings, TP53 mutation has been shown to be a poor prognostic factor in various cancer types (Li et al., 2019a).

Besides N8 and N16 there are other TMEs whose abundance is significantly different across patient types (Supplementary Fig. 17c). Working in ‘discovery’ mode, these TMEs could be used to obtain insights on cohort-differentiating microenvironment features. For instance, neighborhood N4 was associated with poor survival (p-value < 0.0 0 01) and contained Sox9 positive cells, Sox9 having been previously described as an oncogene (Aguilar-Medina et al., 2019).

We next evaluate NaroNet’s ability to capture the heterogeneity of the 35-plex breast cancer cohort by showing the individual interpretable prediction of two patients. The first patient, who survived 33 months, was correctly classified as high risk (RIII) with a prediction confidence of 98.98%. Such prediction was mainly driven by the presence of N4 (i.e., Sox9+, PIR value 1.91) that is highly abundant in this patient (9.07% of the tissue) compared to the average presence of N4 found in the whole patient cohort (1.49%). This is consistent with our global findings that indicate that high risk patients are associated with Sox9 oncogene expression (i.e., neighborhood N4). The second patient, who survived 174 months, was correctly classified as low risk (RI) with a prediction confidence of 93.15%, being the prediction mainly driven by the absence of N8 (i.e., extracellular fibronectin, PIR value 1.84) as its relative abundance is low (0.01%) compared to the average N8 mean abundance (2.21%). Therefore, for this patient, NaroNet correctly quantified a low presence of N8, and associated it to high survival, as it was previously observed cohort-wise (Fig. 6d).

#### Effect of the data input format.

We finally analyzed NaroNet’s ability to predict patient risk subtypes as a function of the input used. On the one hand, as cell segmentation masks are provided along with the public image dataset, cell features were extracted as done in the original reference paper (Jackson et al., 2020). Briefly, a graph of interconnected cells (37-element vectors) was fed to NaroNet, where each cell vector consists of the average expression of the 35 markers plus the cell size and eccentricity. This approach is compared to our proposed strategy based on the use of graphs of patch embeddings. On the other hand, as more than one image was acquired for some patients, we compared the strategy of feeding NaroNet using one graph per image or feeding it with a graph that combines all the images of the patient. We used the same hyperparameters for all the experiments (Supplementary Table 4).

Fig. 6b shows the area under the curve (AUC) for all experiments. As shown, NaroNet achieves the highest prediction performance using a graph containing PCL patches instead of cell masks, and works better when all images of the same patient are combined into a single graph.

## 4. In depth analysis

In this section, we describe additional experiments that were carried out to provide an even more comprehensive understanding of the proposed methods (i.e., NaroNet and PCL). All experiments were conducted using the training protocols introduced in the Results section.

### 4.1. Ablation studies

We examined how NaroNet’s performance varies when selected modules are removed from the original network, specifically phenotype, neighborhood, or area learning. Therefore, we repeated all experiments done with real and synthetic datasets, removing sequentially the phenotype, neighborhood, or area learning modules. The results of the ablation studies are shown in Table 2. Overall, NaroNet performs best when using the three modules. As expected, its performance varies greatly depending on which element of the tumor microenvironment is driving the disease paradigm at hand. For instance, in CCI1, where patient types show distinct cell to cell interactions, the neighborhood learning module is crucial, and when removed, NaroNet is unable to capture cellular interactions, its performance dropping dramatically.

### 4.2. Comparison with other methods

NaroNet is the first WSDL method fully adapted to multiplex imaging. In contrast with other imaging modalities like H&E staining, where WSDL methods can be evaluated in public datasets, e.g., Camelyon16 challenge (Bandi et al., 2018), there is a lack of public multiplex image datasets to objectively evaluate multiplex image analysis frameworks. However, in order to compare NaroNet with other existing approaches that could be applied to multiplex imaging, we adopted two state-of-the-art WSDL methods used to classify H&E tissue sections, adapting them for the analysis of multiplex images from our real cohorts (i.e., Endometrial carcinomas and Breast Cancer cohort).

#### A. CLAM

(Lu et al., 2021) As most WSDL methods, CLAM is based on a two-step strategy. In the first step, the image is divided into image patches (i.e., hundreds of cells) which are fed to a ResNet50 pretrained on ImageNet. In the second step, attention scores are assigned to patch representations considering their relevance in the patient-level classification task at hand. To adapt this method to multiplex imaging, it is necessary to use an alternative patch feature extraction strategy because it is not possible to input multiplex image patches to a RGB-based ResNet50 pretrained on ImageNet. Instead, we used our proposed PCL strategy. To this end, output patch representations from our PCL module were input to CLAM. To choose the size of the image patch we took into consideration how CLAM models patches: CLAM does not try to capture physical interactions between patch representation, but instead models the tumor microenvironment from the information existing within each patch. Therefore, it requires relatively large patch sizes. For this reason, we evaluated CLAM’s performance using two patch sizes, the first following our strategy where one image patch contains one or two cells, and the second, more similar to the original CLAM’s strategy, following the strategy used in (Lu et al., 2021) where one image patch contains dozens of cells.

#### B. Neural Image Compression (NIC)

(Tellez et al., 2019) NIC is also a two-step strategy. In the first step, the image is divided into image patches that are used to train a CNN unsupervisedly. In the second step, feature vectors are arranged to create a compressed image, which is then fed to another CNN (ResNet50) that is trained supervisedly to predict patient-level labels. As done before, we used our PCL strategy to extract features at the cellular level. As NIC is based on a CNN to make patient predictions, thus capturing interactions between patches, we used a small image patch containing one or two cells.

Performance results are provided in Table 3. Compared to CLAM and NIC, NaroNet achieves the best performance in terms of AUC scores. Please note that, besides achieving higher prediction values, our method is inherently interpretable at three levels of complexity (i.e., phenotypes, neighborhoods, and areas).

### 4.3. PCL parameter evaluation

The PCL module learns cellular features from two augmented views of one image crop. Here, as the image crop is $\propto_{L}$ times bigger than the augmented views, the subsequent generated image patches (or views) do not necessarily contain the same pixel information, but can capture information from neighboring pixels. We evaluated NaroNet’s performance using different $\propto_{L}$ values. From the result (Table 4) we can conclude that NaroNet performs better when using a value of $\propto_{L}$ higher than 1. This means that introducing in the learning pipeline information from neighboring pixels is beneficial to extract cellular features.

## 5. Discussion

Our working hypothesis is that relevant elements of the tumor microenvironment can be blindly identified and associated with patient-level tumor information from multiplex imaging data. To this end, we have developed NaroNet, an end-to-end deep learning framework that proves this hypothesis true, as it accurately performs patient predictions from local phenotypes, neighborhoods, and areas that were blindly identified from multiplex immunostained histological data. NaroNet takes advantage of, and improves elements of the two main state-of-the-art computational pathology approaches. From SCA methods, NaroNet inherits the use of graphs to capture phenotype interactions, extending this idea by using GNNs to actually ‘learn’ the most relevant interactions between elements of the tumor microenvironment (Kipf and Welling, 2017). From WSDL, NaroNet uses the concept of learning deep features from patches without the need of manual annotations using only patient-level labels, and applies it to multiplex immunostained sections instead of H&E histological images. Furthermore, instead of being a black-box approach (Rudin, 2019), NaroNet is inherently interpretable as it makes predictions based on the abundances of discovered phenotypes, neighborhoods and areas, thanks to the use of a novel max-sum pooling operation. During the learning process, NaroNet’s parameters are trained to assign patches into never seen TMEs, whose abundances would eventually differentiate patient types. For this reason, NaroNet can be used in ‘discovery mode’ to research new biomarker *signatures* of the tumor biology, or to answer clinically relevant questions, e.g. which tumor features are more predictive of the tumor type or the outcome of the patient. Furthermore, using validated biomarker *signatures*, NaroNet can be trained to provide clinicians with interpretable clinical decisions, since predictions are based on TME annotations which can be mapped back onto the original images. To facilitate individual interpretable predictions, we developed a new metric called predictive influence ratio (PIR) that measures how each tumor microenvironment element contributes to the final prediction.

One of the major bottlenecks in developing high-performance machine learning classifiers for computational pathology is the low number of available labeled tissue images. This is even a greater problem in the case of multi-spectral images, as the use of multiple markers dramatically increases the complexity of the annotation. To address this, we propose a data-efficient contrastive learning loss preprocessing step (PCL). This is a similar strategy to the one followed in state-of-the-art semi-supervised learning frame-works (Chen et al., 2020a; 2020c). These methods learn enriched image representations from large numbers of unlabeled images using an unsupervised deep neural network. Later, a supervised classifier can be trained to obtain outstanding image predictions from small numbers of these enriched, labeled image representations. In our case, all available patient tissue information is divided in patches, i.e., tiles, containing up to two cells, and are introduced in a convolutional neural network to create self-supervised low-dimensional enriched embeddings of these patches. These embeddings allow for comprehensive discrimination of pixel-level features such as, cell morphology, marker intensity, marker colocalization, etc. thus reducing the inherent heterogeneity existing within and between tissues. We hypothesized that the use of these prototypical enriched representations extracted from the images might help with the classification of low number of patients represented by them. Furthermore, the volume of data space is decreased so that NaroNet’s computational time is reduced allowing the use of architecture search algorithms that would ultimately increase predictive performance.

We have validated NaroNet using both synthetic and real data. Using a novel multiplex tissue image simulator we created realistic patient cohorts with tunable presence of specific TMEs, providing an ideal objective benchmark to test the performance of the system. Indeed, our extensive validation using synthetic data successfully confirms that NaroNet can learn relevant TMEs - local phenotypes, cell-interaction neighborhoods, and neighborhood-interaction areas -, even when their presence in the tissue is rare. Using a high-grade endometrial carcinoma patient cohort, NaroNet found, among other TMEs that could be explored, a local phenotype expressing CD8, PD1, and FOXP3 whose high abundance was associated with the POLE mutation, while achieving a prediction accuracy of 93.75%. This finding is in accordance with what has already been described in the literature. Moreover, we confirmed using a semi-automated computational pathology software (QuPath), that the abundance of this specific phenotype correlates positively with the one found by NaroNet. This nicely shows that NaroNet can be a useful tool in research environments, as it can help to blindly identify novel TMEs that are related to the biology of the tumor. Using a public breast cancer dataset, NaroNet found TMEs that were associated with the patient’s survival achieving an AUC of 0.73. Strikingly, NaroNet did not require human supervision to learn, from a pool of millions of cells stained by 35 markers, decisive neighborhoods consisting of cells expressing Sox9 and extracellular fibronectin, respectively, that were related to the survival of the patient. In addition, we show that NaroNet performs better when fed with an enriched graph created from image patches than when using cell features obtained from cell segmentation masks. This shows that NaroNet is able to learn relevant tumor microenvironmental information without the highly demanding task of segmenting all cells in the tissue. We also prove that using graphs to represent patients is more advantageous than using images, as patient’s stained tissue sections can be combined together into a single, disjoint graph providing NaroNet with more information to make better predictions.

Finally, we have presented an ablation study that shows that the three levels of spatial complexity used by NaroNet to model the tumor microenvironment (i.e., local phenotypes, neighborhoods and areas) contribute individually to achieve better predictions. Moreover, a comparison with two state-of-the-art WSDL methods shows that NaroNet is able to achieve more accurate predictions while providing an inherent interpretability of the reason behind those predictions that the rest of the methods lack.

## 6. Conclusion

We have presented NaroNet, and ensemble of networks that unsupervisedly identifies and annotates relevant TMEs that drive patient outcomes. Since we have shown that NaroNet is able learn *in situ* highly predictive TMEs that confirm the existing literature, it is possible to affirm that the analysis of new predictive TMEs discovered by NaroNet could provide novel insights into the mechanisms of disease progression. This could be used in clinical settings, and more importantly, it makes NaroNet a valuable research tool for the discovery of novel biomarkers. Furthermore, the fact that NaroNet’s clinical predictions are directly based on the annotations of TMEs results in an important breakthrough in computational pathology, as it contributes to the whitening of DL black-boxes. Indeed, our model allows clinicians to understand which TMEs drive the prediction of each patient safely and reliably since DL neuron activations are related to specific biological structures that can be mapped back into the original images. Therefore, NaroNet could be an optimal solution for the rapid clinical translation of biomarker discovery *signatures*, where DL models trained to quantify relevant TMEs are then applied to new incoming patients by providing clinicians with interpretable predictions.

### Implementation details

Patch contrastive learning is implemented in Python 3.7.3 using Tensorflow 1.14.0. NaroNet is implemented in Python 3.7.3 using PyTorch 1.4.0. Architecture search was performed using ray 1.0.0 (Liaw et al., 2018) and hyperpopt 0.2.3. Synthetic datasets were generated in MATLAB v2019b. Python libraries that were also used include imgaug 0.4.0, tqdm 4.48.2, scipy 1.5.4, numpy 1.18.2, sklearn 0.23.2, seaborn 0.11.0, and pandas 1.1.1. All the experiments were carried out using a server with 16 Intel(R) Xeon(R) E5–2623 v3 @ 3.00GHz CPUs, a RAM of 256 GBs, and 4 GeForce RTX 2080 Ti GPUs of 11GBs. For use as a framework, NaroNet’s source code is available on GitHub ( https://github.com/djimenezsanchez/NaroNet ).

All 3 datasets used in this study are publicly available and can be accessed online. All synthetic patient cohorts (including multiplex images, ground-truth masks, and patient data) and high-grade endometrial cancer cohorts (including multiplex images and patient data) are available at Zenodo (https://doi.org/10.5281/zenodo.4596337). Breast cancer cohort is publicly available from the original authors (Jackson et al., 2020) at Zenodo (https://doi.org/10.5281/zenodo.3518284).

## Supplementary Material

Supplementary material

## Figures and Tables

**Table 1:** NaroNet performance: synthetic experiments. NaroNet’s classification accuracy (and 95% confidence interval) and interpretability calculated as the intersection of the most relevant extracted TME and the ground-truth of each synthetic experiment. Legend. PMI: phenotype marker expression; PF: phenotype frequency; CCI: cell-cell interaction, and NNI for neighborhood-neighborhood interaction. Index 1 refers to moderate presence, and index 2 to rare presence.

Metric / Disease Paradigm	PMI1	PMI2	PF1	PF2	CCI1	CCI2	NNI1
**Accuracy (% ± Cl 95%)**	93.618.9	99.710.3	99.710.3	78.113.9	98.911.0	47.815.1	86.712.0
**Interpretability (%)**	92.42	28.41	81.36	51.08	92.89	72.80	63.08

**Contrast Accuracy (%)**	59.1	71.0	58.7	55.2	52.8	58.2	78.6

**Table 2:** Ablation study on the synthetic and real experiments. Bold-faced results highlight the best performing result. AUCs are listed for the experiments with real patient cohorts. The synthetic experiments list the accuracy values.

Ablation study	Endometrial Cancer (AUC)				Breast Cancer (AUC)			Synthetic experiments (Acc.)			
	POLE	Hist.	MMR	CNV	RI-RIII	RI-RII	RII-RIII	PMI1	PF1	CCI1	NNI1
All networks	**0.98**	**0.98**	0.97	0.97	**0.73**	**0.68**	**0.56**	93.6	**99.7**	**98.9**	**86.7**

w/o Phenotype learning	**0.98**	**0.98**	**0.99**	0.98	0.66	0.65	0.56	77.7	98.8	96.7	**88.9**
w/o Neighborhood learning	0.94	0.96	0.98	0.95	0.63	0.62	0.52	60.3	63.5	35.3	60.2
w/o Area learning	0.93	0.97	**0.99**	**0.99**	0.70	0.65	0.52	**98.8**	80.8	98.6	86.5

**Table 3:** Comparison of NaroNet with other WSDL methods. AUC scores obtained using three weakly-supervised methods over a 10 fold cross validation, for the endometrial carcinomas and breast cancer datasets. A small patch size corresponds to 15×15 and 18×18 pixels, and a large patch size is 90×90 and 100×100 pixels in the endometrial carcinomas and breast cancer datasets, respectively.

Methods	Patch size	Endometrial Cancer				Breast Cancer		
		POLE	Hist.	MMR	CNV	RI-RIII	RI-RII	RII-RIII
NaroNet	Small	**0.98**	**0.98**	**0.97**	**0.97**	**0.73**	**0.68**	**0.56**

CLAM	Small	0.91	0.95	0.92	0.89	0.60	0.57	0.48
	Big	0.95	0.92	0.87	0.86	0.59	0.57	0.49

NIC	Small	**0.98**	**0.98**	**0.97**	0.94	0.57	0.55	0.47

**Table 4:** Study of the effect of the image crop size for PCL.

	Endometrial Cancer				Breast Cancer		
	POLE	Hist.	MMR	CNV	RI-RIII	RI-RII	RII-RIII
α_L_ = 1	**0.98**	0.95	0.95	0.95	0.57	0.56	0.48
α_L_ = 1.15	**0.98**	**0.98**	**0.97**	**0.97**	**0.73**	**0.68**	**0.56**
α_L_ = 1.30	0.97	0.94	0.95	0.92	0.50	0.48	0.49
