# Supplementary material for: NaroNet: Discovery of tumor microenvironment elements from highly multiplexed images
Source: Med Image Anal. Author manuscript; Available in PMC 2023 Feb 28. (PMC9972483; doi:10.1016/j.media.2022.102384)
Supplement: Supplementary material [file NIHMS1846804-supplement-Supplementary_material.zip › mmc1.docx]

**Supplementary Materials of “NaroNet: Discovery of tumor microenvironment elements from highly multiplexed imaging”**

Daniel Jiménez-Sánchez^1^*^,^*^2^, Mikel Ariz^1^, Hang Chang^2^, Xavier Matias-Guiu^3^ , Carlos E. de Andrea^4^ & Carlos Ortiz-de-Solórzano^1,*^

^1^*Solid Tumors and Biomarkers Program, IDISNA, and Ciberonc, Center for Applied Medical Research, University of Navarra, 31008, Pamplona, Spain*

^2^*Biological Systems and Engineering Division, Lawrence Berkeley National Laboratory, CA, 94720, Berkeley, USA*

^3^*Department of Pathology, Hospital U Arnau de Vilanova and Hospital U de Bellvitge, Universities of Lleida and Barcelona, IDIBELL, IRBLLEIDA, Ciberonc, Lleida, 25198, Spain*

^4^*Department of Pathology, IDISNA, Ciberonc, Clínica Universidad de Navarra, University of Navarra, 31008, Pamplona, Spain*

^*^*Corresponding author*

**Supplementary Materials: Appendix A. High grade Endometrial carcinomas.**

**Dataset description.** Formalin-fixed, paraffin-embedded (FFPE) samples from a retrospective collection of 12 high-grade endometrial carcinomas were analyzed, including 9 FIGO grade 3 endometrioid carcinomas and 3 serous-like carcinomas. Cases were collected from the surgical pathology files of Hospital universitary Bellvitge. Pathologic and molecular classification was performed at the Hospital universitary Bellvitge (IDIBELL, Barcelona, Spain) and Instituto de Investigación Biomédica de Lleida (IRBLLEIDA, Lleida, Spain). All patients signed informed consent to use the excedent of pathologic analysis for research, and the study was carried out after approval from the IRBLLEIDA Investigation Committee protocol numbers: CEIC-2083. All cases in the cohorts were reviewed by a local pathologist using hematoxylin and eosin–stained preparations and the tumor histology variant was confirmed by morphology analysis. All cases have been molecularly classified in previous studies (León-Castillo et al., 2020). The microsatellite instability (MSI) subclass was determined by immunohistochemistry for DNA mismatch repair (MMR) proteins. Genomic copy number variation was determined using an array comparative genomic hybridization (aCGH) on sample pairs as previously described (Miller et al., 2020). POLE variants were studied by Sanger sequencing or NGS of the complete exonuclease domain (exons 9–14) or targeted sequencing of exons 9, 13, and 14. Tissue sections from twelve Formalin-fixed, paraffin-embedded (FFPE) high-grade endometrial carcinomas were stained with a seven-color multiplex panel targeting key elements of the immune environment: CD4+ and CD8+ T cells, the transcription factor FOXP3, the bona fide T cell activation marker CD137 (4-1BB), the programmed cell death-1 (PD-1), cytokeratin, and nuclear detection with DAPI. Whole-tissue sections were scanned using a Vectra-Polaris Automated Quantitative Pathology Imaging System (Perkin Elmer Inc., Waltham, MA, USA). Clinicopathological patient-level information was available for these tumors (León-Castillo et al., 2020), including the microsatellite instability (MSI) subclass, genomic copy number variation, and POLE mutation variants.

**Multiplex panel and staining protocol.** The exploration of the immune environment of high-grade endometrial carcinomas was done using a previously developed and validated seven-color multiplex panel targeting CD4+ and CD8+ T cells, the transcription factor FoxP3, the bona fide T cell activation marker CD137 (4-1BB), the programmed cell death-1 (PD-1), cytokeratin, and nuclear detection with DAPI. Multiplex immunofluorescence panel was used to enable the simultaneous examination of several cellular markers on a single formalin-fixed paraffin-embedded (FFPE) tissue section. Multiplex immunofluorescence development and validation workflow and protocols have been implemented as previously described (Abengozar-Muela et al., 2020a; Salas-Benito et al., 2021; Schalper et al., 2019). Briefly explaining, 5 µm sections of FFPE tissue were deparaffinized and antigen retrieval was performed using DAKO PT-Link heat-induced antigen retrieval with low pH (pH6) or high pH (pH 9) target retrieval solution (DAKO). Each tissue section was subjected to five successive rounds of antibody staining, each round consisting of protein blocking with antibody diluent/block (Akoya Biosciences ARD1001EA), incubation with primary antibody, Opal Polymer anti-mouse/rabbit HRP (Akoya Biosciences ARH1001EA), followed by tyramide signal amplification (TSA) with Opal fluorophores (Akoya Biosciences) diluted 1:100 in 1X plus amplification diluent (Akoya Biosciences FP1498). The primary antibody panel included: CD4 (Mouse monoclonal, clone 4B12, ready-to-use, Agilent, product number IS64930-2), CD8 (Mouse monoclonal, clone C8/144B, ready-to-use, Agilent, product number GA62361-2), FoxP3 (Mouse monoclonal, clone 236A/E7, 1:300, Abcam, product number ab20034), PD1 (Mouse monoclonal, clone NAT105, ready-to-use, Cell Marque, product number 315M), CD137 (TNFRSF9 or 4-1BB, Mouse monoclonal, clone BBK-2, 1:80, ThermoFisher, product number MA5-13736), cytokeratin (Mouse monoclonal, clone AE1/AE3, ready-to-use, Leica Biosystems, product number NCL-L-AE1/AE). Finally, in the last round, nuclei were counterstained with spectral DAPI (Akoya Biosciences FP1490) and sections mounted with Faramount Aqueous Mounting Medium (Dako S3025).

**Supplementary Materials: Appendix B. Architecture search.**

To determine the configuration that provides the highest predictive and interpretability performance, we incorporate an architecture search algorithm into the training phase of NaroNet (Kyriakides and Margaritis, 2020). This is feasible since, by our choice of using PCL, the large multispectral input images are embedded in compact, low dimensional enriched patch-graphs. Due to this, NaroNet does not require very deep, multilayer neural networks to successfully classify patients. This reduces drastically the training time and allows an architecture search to iteratively optimize the parameters of the network based on the training data. We choose the Tune framework to perform architecture search (Liaw et al., 2018), which requires defining three main modules (Elsken et al., 2018). A search space, which in our case is the set of interchangeable modules (see Supplementary Materials: Appendix B), the loss functions, and hyperparameters. A search strategy is used to explore the search space, i.e., ASHA (Li et al., 2020), which works by the repetition of three simple steps: *i.* train a considerable number of architectures with a few numbers of epochs, *ii.* evaluate all configurations, *iii.* keep the top η architectures, *iv.* increase the number of epochs to train the architectures, *iv.* and repeat the process until only one architecture is left. A performance estimation strategy which in our case is based on the classification accuracy of the model on unseen data -valid both for synthetic and real data-, and the interpretability of the model, only for the synthetic data for which an absolute ground truth exists. To this end, the network is trained using a training set containing 90% of the data, and the performance is evaluated on the validation set that contains the remaining 10% of the data (i.e., one fold from a 10-fold cross-validation configuration).

**Supplementary Materials: Appendix C. Architectural variations.**

From the previously explained baseline NaroNet architecture, some variations have been proposed and implemented that are selected after a process of architecture optimization (Supplementary Materials: Appendix A). The architectural variations are described in the next paragraphs:

**1. Multiple TME assignment.** In classical computational biology, a cell or tissue element is commonly assigned to a single TME (Codeluppi et al., 2018) (Levine et al., 2015). Here we allow patches to be assigned to more than one TME, e.g. patches containing elongated Ki67 positive cells can be assigned to two phenotypes, the first capturing cells with an elongated morphology, and the second cells expressing Ki67. To this end, we eliminate the max operator in **eqs. 6, 7, 8**.

**2. Leveraging patch relevance.** Following the strategy behind the standard semi-quantitative analysis of immunostained sections, such as the H-score (Villalba et al., 2019), where cells or tissue elements are assigned a semi-quantitative factor to measure the abundance of TMEs, we also allow our neural networks to assign different probability weights to each patch in a given TME. To this end, we replace the *softmax* operator in **eqs. 6, 7, 8** with a *sigmoid* operator. This way neural activations in **eqs. 6, 7, 8** may add up to values higher than 1, thus eliminating the limitation imposed by the *softmax* operator.

**3. Global reasoning unit.** Multiplex image staining quality can be affected by many factors: the presence of autofluorescence, spectral leakage between different biomarkers, low expression of some antigens, etc., leading to a great deal of variability within and between samples. When hundreds of samples are available, it is possible to ‘learn’ some of the technical variabilities present in the tissue. Here, we use a global relation reasoning unit (GloRe) (Chen et al., 2019) to capture relations between disjoint and distant regions. To this end, as we preprocess the input of NaroNet, patch embeddings are fed to this processing unit, which returns the patch embeddings with the same shape, but with their features being leveraged by the rest of the tissue. This way, the processing unit can use distant regions and normalize their image information.

**4. Alternative GNN architectures** Several GNN architectures have been proposed such as residual networks (ResNet) (Zhang and Meng, 2019), jumping knowledge networks (JKNet) (Xu et al., 2018), and IncepNet (Kazi et al., 2019). We have compared these three alternative architectures to determine the one that provides better learning performance.

**5. Data augmentation for Graph Neural Networks.** Data augmentation expands the training dataset to improve the model’s generalization. Given a graph $\mathcal{G=}\left( \mathcal{Z,A} \right)$, augmentation operations are carried out sequentially. We investigate which approaches provide a better generalization of patient-outcome predictions and include a tunable parameter $\rho$ that weighs the number of edges/nodes that are being augmented. The most commonly used augmentation operation in GNNs is to drop $\rho\times L$ edges randomly in each training epoch (Rong et al., 2019). We also propose to add $\rho\times L$ edges randomly to the graph. Additionally, we mask out node embedding information from $\rho\times L$ nodes.

**Supplementary Materials: Appendix D. Interpretability performance measure.**

In those cases when ground-truth masks are available (e.g., synthetic experiments) referring to regions that drive the disease paradigm, we evaluate whether the proposed method classified patients correctly based on them or not. To this end, an interpretability performance measure is proposed to objectively quantify NaroNet’s interpretability. The process is as follows, first, we use the differential TME analysis to extract which TMEs are significant when making patient predictions. Second, we create a binary image, $I_{TME}$, with the patches that were assigned to the significant TMEs. This mask is then compared with the ground-truth mask, $I_{GT}$. Next, we calculate the intersection between both binary images, and divide the result by the number of pixels present in $I_{TME}$:

$$interpretability=\frac{I_{TME}\cap I_{GT}}{sum\left( I_{TME} \right)}$$

(1)

This way, we calculate the ratio of pixels that were relevant in NaroNet’s prediction that lied in the regions of the ground-truth mask that contain the disease paradigm. Here, an interpretability value of 1 means that NaroNet’s most relevant TME is completely contained in the ground truth mask. This process is repeated for each image/patient in the cohort providing a mean value of interpretability.

**Supplementary Materials: Appendix E. Image analysis with Qupath.**

Image analysis was performed on whole-tissue sections using the open-source digital pathology software QuPath (Bankhead et al., 2017) version 0.2.3, as previously described (Abengozar-Muela et al., 2020b). In short, cell segmentation based on nuclear detection was performed using StarDist 2D algorithm, a method that localizes nuclei via star-convex polygons, incorporated into QuPath software by scripting. A random trees classifier was trained separately for each cell marker by an experienced pathologist annotating the tumor regions. Interactive feedback on cell classification performance, provided during training in the form of markup image, improved the accuracy of machine learning-based phenotyping. All phenotyping and subsequent quantifications were performed blinded to the sample identity. Cells close to the border of the images were removed to reduce the risk of artifacts. Based on the fluorescence panels, cells were classified as CD4+ and CD8+ T cells. Subpopulations within CD4+ and CD8+ T cells were then subclassified by the presence and absence of three additional markers: FOXP3, PD1, and CD137. The staining pattern for CD137 considered as positive was described earlier (Alfaro et al., 2015). Cells negative for these markers were defined as “other cell types”.

**References**

Abengozar-Muela, M., Esparza, M.V., Garcia-Ros, D., Vásquez, C.E., Echeveste, J.I., Idoate, M.A., Lozano, M.D., Melero, I., de Andrea, C.E., 2020a. Diverse immune environments in human lung tuberculosis granulomas assessed by quantitative multiplexed immunofluorescence. Mod. Pathol. 33, 2507–2519. https://doi.org/10.1038/s41379-020-0600-6

Abengozar-Muela, M., Esparza, M.V., Garcia-Ros, D., Vásquez, C.E., Echeveste, J.I., Idoate, M.A., Lozano, M.D., Melero, I., de Andrea, C.E., 2020b. Diverse immune environments in human lung tuberculosis granulomas assessed by quantitative multiplexed immunofluorescence. Mod. Pathol. 33, 2507–2519. https://doi.org/10.1038/s41379-020-0600-6

Alfaro, C., Echeveste, J.I., Rodriguez-Ruiz, M.E., Solorzano, J.L., Perez-Gracia, J.L., Idoate, M.A., Lopez-Picazo, J.M., Sanchez-Paulete, A.R., Labiano, S., Rouzaut, A., Oñate, C., Aznar, A., Lozano, M.D., Melero, I., 2015. Functional expression of CD137 (4-1BB) on T helper follicular cells. Oncoimmunology 4. https://doi.org/10.1080/2162402X.2015.1054597

Bankhead, P., Loughrey, M.B., Fernández, J.A., Dombrowski, Y., McArt, D.G., Dunne, P.D., McQuaid, S., Gray, R.T., Murray, L.J., Coleman, H.G., James, J.A., Salto-Tellez, M., Hamilton, P.W., 2017. QuPath: Open source software for digital pathology image analysis. Sci. Rep. 7. https://doi.org/10.1038/s41598-017-17204-5

Chen, Y., Rohrbach, M., Yan, Z., Shuicheng, Y., Feng, J., Kalantidis, Y., 2019. Graph-based global reasoning networks. Proc. IEEE Comput. Soc. Conf. Comput. Vis. Pattern Recognit. 2019, 433–442. https://doi.org/10.1109/CVPR.2019.00052

Codeluppi, S., Borm, L.E., Zeisel, A., La Manno, G., van Lunteren, J.A., Svensson, C.I., Linnarsson, S., 2018. Spatial organization of the somatosensory cortex revealed by osmFISH. Nat. Methods 15, 932–935. https://doi.org/10.1038/s41592-018-0175-z

Elsken, T., Metzen, J.H., Hutter, F., 2018. Neural architecture search: A survey. arXiv Prepr. arXiv 1808.05377.

Kazi, A., Shekarforoush, S., Arvind Krishna, S., Burwinkel, H., Vivar, G., Kortüm, K., Ahmadi, S.A., Albarqouni, S., Navab, N., 2019. InceptionGCN: Receptive Field Aware Graph Convolutional Network for Disease Prediction. Lect. Notes Comput. Sci. (including Subser. Lect. Notes Artif. Intell. Lect. Notes Bioinformatics) 11492 LNCS, 73–85. https://doi.org/10.1007/978-3-030-20351-1_6

Kyriakides, G., Margaritis, K., 2020. An Introduction to Neural Architecture Search for Convolutional Networks. arXiv Prepr. arXiv 2005.11074.

León-Castillo, A., Gilvazquez, E., Nout, R., Smit, V.T.H.B.M., McAlpine, J.N., McConechy, M., Kommoss, S., Brucker, S.Y., Carlson, J.W., Epstein, E., Rau, T.T., Soslow, R.A., Ganesan, R., Matias-Guiu, X., Oliva, E., Harrison, B.T., Church, D.N., Gilks, C.B., Bosse, T., 2020. Clinicopathological and molecular characterisation of ‘multiple-classifier’ endometrial carcinomas. J. Pathol. 250, 312–322. https://doi.org/10.1002/path.5373

Levine, J.H., Simonds, E.F., Bendall, S.C., Davis, K.L., Amir, E.A.D., Tadmor, M.D., Litvin, O., Fienberg, H.G., Jager, A., Zunder, E.R., Finck, R., Gedman, A.L., Radtke, I., Downing, J.R., Pe’er, D., Nolan, G.P., 2015. Data-Driven Phenotypic Dissection of AML Reveals Progenitor-like Cells that Correlate with Prognosis. Cell 162, 184–197. https://doi.org/10.1016/j.cell.2015.05.047

Li, L., Jamieson, K., Rostamizadeh, A., Gonina, E., Ben-tzur, J., Hardt, M., Recht, B., Talwalkar, A., 2020. A System for Massively Parallel Hyperparameter Tuning. Proc. Mach. Learn. Syst. 2, 230–246.

Liaw, R., Liang, E., Nishihara, R., Moritz, P., Gonzalez, J.E., Stoica, I., 2018. Tune: A research platform for distributed model selection and training. arXiv Prepr. arXiv 1807.05118.

Miller, R.E., Leary, A., Scott, C.L., Serra, V., Lord, C.J., Bowtell, D., Chang, D.K., Garsed, D.W., Jonkers, J., Ledermann, J.A., Nik-Zainal, S., Ray-Coquard, I., Shah, S.P., Matias-Guiu, X., Swisher, E.M., Yates, L.R., 2020. ESMO recommendations on predictive biomarker testing for homologous recombination deficiency and PARP inhibitor benefit in ovarian cancer. Ann. Oncol. 31, 1606–1622. https://doi.org/10.1016/j.annonc.2020.08.2102

Rong, Y., Huang, W., Xu, T., Huang, J., 2019. DropEdge: Towards the very deep graph convolutional networks for node classification. arXiv Prepr. arXiv 1907.10903.

Salas-Benito, D., Conde, E., Tamayo-Uria, I., Mancheño, U., Elizalde, E., Garcia-Ros, D., Aramendia, J.M., Muruzabal, J.C., Alcaide, J., Guillen-Grima, F., Minguez, J.A., Amores-Tirado, J., Gonzalez-Martin, A., Sarobe, P., Lasarte, J.J., Ponz-Sarvise, M., De Andrea, C.E., Hervas-Stubbs, S., 2021. The mutational load and a T-cell inflamed tumour phenotype identify ovarian cancer patients rendering tumour-reactive T cells from PD-1+ tumour-infiltrating lymphocytes. Br. J. Cancer 1–12. https://doi.org/10.1038/s41416-020-01218-4

Schalper, K.A., Rodriguez-Ruiz, M.E., Diez-Valle, R., López-Janeiro, A., Porciuncula, A., Idoate, M.A., Inogés, S., de Andrea, C., López-Diaz de Cerio, A., Tejada, S., Berraondo, P., Villarroel-Espindola, F., Choi, J., Gúrpide, A., Giraldez, M., Goicoechea, I., Gallego Perez-Larraya, J., Sanmamed, M.F., Perez-Gracia, J.L., Melero, I., 2019. Neoadjuvant nivolumab modifies the tumor immune microenvironment in resectable glioblastoma. Nat. Med. 25, 470–476. https://doi.org/10.1038/s41591-018-0339-5

Villalba, M., Exposito, F., Pajares, M.J., Sainz, C., Redrado, M., Remirez, A., Wistuba, I., Behrens, C., Jantus-Lewintre, E., Camps, C., Montuenga, L.M., Pio, R., Lozano, M.D., de Andrea, C., Calvo, A., 2019. TMPRSS4: A Novel Tumor Prognostic Indicator for the Stratification of Stage IA Tumors and a Liquid Biopsy Biomarker for NSCLC Patients. J. Clin. Med. 8, 2134. https://doi.org/10.3390/jcm8122134

Xu, K., Li, C., Tian, Y., Sonobe, T., Kawarabayashi, K.I., Jegelka, S., 2018. Representation learning on graphs with jumping knowledge networks, in: 35th International Conference on Machine Learning, ICML 2018. pp. 8676–8685.

Zhang, J., Meng, L., 2019. Gresnet: Graph Residual Network for Reviving Deep Gnns From Suspended Animation. arXiv. Prepr. arXiv 1909.05729.

**Supplementary Figure 1:** Visualization of the Phenotype Marker Intensity (PMI1) disease paradigm. **a-c.** Multiplex images obtained from the tissue simulator that correspond to three patients, each of the patients corresponding to a distinct type. **d-f.** Image crop extracted from (a-c). **g-i.** Ground-truth mask where each color corresponds to a different cell phenotype. **j-l.** Ground-truth mask where each color corresponds to a different neighborhood, which is an agglomeration of cells. **m.** Legend of colors that refer to their corresponding phenotypes. **n.** Legend of colors that refer to their corresponding neighborhoods. **o.** Marker expression of Phenotype 6 for three patient typologies (I, II, and III). Note that the expression of Marker 6 in Phenotype 6 varies with the patient typology.

**Supplementary Figure 2:** Visualization of the Phenotype Frequency (PF1) Disease Paradigm. **a-c.** Multiplex images obtained from Synplex that correspond to three patients, each patient corresponding to one type. **d-f.** Image crop extracted from (a-c). **g-i.** Ground-truth mask where each color corresponds to a different cell phenotype. **j-l.** Ground-truth mask where each color corresponds to a different neighborhood, which is an agglomeration of cells. **m.** Legend of colors that refer to their corresponding phenotypes. **n.** Legend of colors that refer to their corresponding neighborhoods. **o.** Relative abundance of phenotypes for three distinct patient typologies (I, II and III). Note that the relative abundance of Phenotype 6 is different for each patient typology.

**Supplementary Figure 3:** Visualization of the Cell-Cell Interaction (CCI1) disease paradigm. **a-c.** Multiplex images obtained from Synplex that correspond to three patients, each patient corresponding to one type. **d-f.** Image crop extracted from (a-c). **g-i.** Ground-truth mask where each color corresponds to a different cell phenotype. **j-l.** Ground-truth mask where each color corresponds to a different neighborhood, which is an agglomeration of cells. **m.** Legend of colors that refer to their corresponding phenotypes. **n.** Legend of colors that refer to their corresponding neighborhoods. **o.** Cell-cell interactivity for three distinct patient typologies (I, II and III). Note that the interaction of phenotypes 4 and 5 is different for each patient typology.

**Supplementary Figure** **4:** Visualization of the neighborhood-neighborhood interaction (NNI) disease paradigm. **a-c.** Multiplex images obtained from Synplex that correspond to three patients, each patient corresponding to one type. **d-f.** Image crop extracted from (a-c). **g-i.** Ground-truth mask where each color corresponds to a different cell phenotype. **j-l.** Ground-truth mask where each color corresponds to a different neighborhood, which is an agglomeration of cells. **m.** Legend of colors that refer to their corresponding phenotypes. **n.** Legend of colors that refer to their corresponding neighborhoods. **o.** Interaction between neighborhoods for three distinct patient typologies (I, II and III). Note that the interaction between neighborhood 2 and 3 is different with respect to the patient typology.

| **Grid search (specific to NaroNet)** | | | | | | | | | | | | | | | | | | |
| --- | --- | --- | --- | --- | --- | --- | --- | --- | --- | --- | --- | --- | --- | --- | --- | --- | --- | --- |
| Number of phenotypes | 4 | | | 7 | | | | | | | | | | 10 | | | | |
| Number of neighborhoods | 3 | | | 6 | | | | | | | | | | 9 | | | | |
| Number of areas | 2 | | | 5 | | | | | | | | | | 8 | | | | |
| Global reasoning unit (GLORE) | Activated | | | | | | | | | | Deactivated | | | | | | | |
| Patch activation function | Softmax | | | | | | | | | | Sigmoid | | | | | | | |
| Max-pooling on patch assignment | Activated | | | | | | | | | | Deactivated | | | | | | | |
| Orthogonal loss | Activated | | | | | | | | | | Deactivated | | | | | | | |
| Patch entropy loss | Activated | | | | | | | | | | Deactivated | | | | | | | |
| Patient entropy loss | Activated | | | | | | | | | | Deactivated | | | | | | | |
| MinCut entropy loss | Activated | | | | | | | | | | Deactivated | | | | | | | |
| Lasso loss (last layers) | Activated | | | | | | | | | | Deactivated | | | | | | | |
| Data augmentation (in %) | 0.01% | | 0. 1% | | | 1% | | | | | | 5% | | | | Deactivated | | |
| Graph convolution unit | ResNet | | IncepNet | | | | | | | | | | | | | | JKNet | |
| Number of hops in GNN | 1 | | | | 2 | | | | | | | | | | 3 | | | |
| Patch activation threshold | 0 | | 0.2 | | | | 0.4 | | | | | | | | | | | 0.6 |
|  | | | | | | | | | | | | | | | | | | |
| **Grid search (ML standard parameters)** | | | | | | | | | | | | | | | | | | |
| Batch size | 2 | 6 | | | 12 | | | | | | | | 16 | | | | | 20 |
| Weight decay | 0.0001 | 0.001 | | | 0.01 | | | | | | | | 0.1 | | | | | 1 |
| Learning rate | 0.0001 | 0.001 | | | | | | | | 0.01 | | | | | | | | 0.1 |
| Dropout rate | 5% | 10% | | | | | 15% | | | | | | 20% | | | | | 25% |
| Hidden layers | 32 | | 44 | | | | | 64 | | | | | 96 | | | | | 128 |
| Normalize patch embeddings (x-axis) | Activated | | | | | | | | Deactivated | | | | | | | | | |
| Normalize patch embeddings (y-axis) | Activated | | | | | | | | Deactivated | | | | | | | | | |
| Batch normalization | Activated | | | | | | | | Deactivated | | | | | | | | | |

**Supplementary Table 1:** Architecture search space used by NaroNet. Parameter configurations specific to NaroNet are specified above, and parameter configurations that are common to most ML-based classifiers are described below.

| **Optimal parameters (specific to NaroNet)** | | | |
| --- | --- | --- | --- |
| Number of phenotypes | 10 | Number of neighborhoods | 9 |
| Number of areas | 8 | Global reasoning unit (GLORE) | Deactivated |
| Patch activation function | False | Max-pooling on patch assignment | Softmax |
| Orthogonal loss | Deactivated | Patch entropy loss | Activated |
| Intra-TME variability function | Activated | Patch activation threshold | 0 |
| Patient entropy loss | Deactivated | Mincut entropy loss | Activated |
| Lasso loss (last layers) | Deactivated | Graph convolution unit | Resnet |
| Data augmentation (in %) | 0.01% | Number of hops in GNN | 3 |
|  | | | |
| **Optimal parameters (ML standard parameters)** | | | |
| Batch size | 6 | Weight decay | 0.001 |
| Learning rate | 0.001 | Dropout rate | 20% |
| Hidden layers | 44 | Normalize patch embeddings (y-axis) | False |
| Normalize patch embeddings (x-axis) | False | Batch normalization | True |

**Supplementary Table 2:** NaroNet optimized parameters after 500 runs in CCI1 disease paradigm experiment. Parameter configurations specific to NaroNet are specified above, and below, configuration parameters that are common to most ML-based classifiers.

**Supplementary Figure 5:** Boxplot evaluation of individual NaroNet parameters with respect to the test loss and the interpretability accuracy for the CCI1 disease paradigm in 500 architecture variations. **a-e.** Proposed and state-of-the-art unsupervised losses. **f-i.** Proposed and state-of-the-art units. P-value annotation legend: ns: p>0.05, *: p<0.05, **: p<0.01, ***: p<0.001, ****: p<0.0001.

**Supplementary Figure 6:** Boxplot evaluation of individual NaroNet parameters with respect to the test loss and the interpretability accuracy for the CCI1 disease paradigm in 500 architecture variations. **a-e.** Proposed cell type assignment parameters. **f-i.** Parameters typically found in machine learning pipelines. P-value annotation legend: ns: p>0.05, *: p<0.05, **: p<0.01, ***: p<0.001, ****: p<0.0001.

**Supplementary Figure 7**: Test performance of CCI1 disease paradigm experiment with optimized architectural parameters. **a,d.** ROC and recall curves comparing patient type I vs. type II. **b,e.** ROC and recall curves comparing patient type I vs. type III. **c,f.** ROC and recall curves comparing patient type II vs. type III. **e.** Training-validation accuracy curve. **h.** Training-validation loss curve. **i.** Training-validation patch entropy loss. **j.** Confusion matrix.

**Supplementary Figure 8**: Neighborhoods learned by NaroNet in CCI1 synthetic experiment. **a.** Color legend for each marker. **b.** Heatmap showing the z-scored mean marker expression, coloured by phenotype **c.** Absolute patch counts of each phenotype in the cohort. **d-l.** Left, representative 9 pixel patches being the central one assigned to a specific neighborhood type, and right, histogram showing the certainty distribution of patches assigned to a neighborhood according to patch entropy loss.

**Supplementary Figure 9**: TME composition of image fields in CCI1 synthetic experiment. **a.** Heatmap showing the sum-to-1 normalized distribution of neighborhoods in 240 images/patients, coloured by label (Patient type). Both images/patients and neighborhoods are ordered by a hierarchical clustering.

**Supplementary Figure 10**: TME differential composition analysis for CCI1 synthetic experiment. **a.** Table showing the most relevant inferred neighborhoods by NaroNet and its corresponding p-value in a Mann-Whitmann test. **b-e**. Violinplots showing the relative abundance of N3, N7, N9, and N1 neighborhoods for each patient group, coloured by patient type.

**Supplementary Figure 11**: Illustration of most relevant TMEs to classify patients in the synthetic CCI1 disease paradigm. **a.** Heatmap showing N3, N9, N1, N7, and N8 PIR values for each patient in the cohort. The higher the PIR value, the higher is the relevance of the TME for a specific patient. **b.** Color legend. **c-d.** Left, pixel image from a patient correctly classified to its corresponding label along with squares that represent patches assigned to the most relevant TME in the patient’s prediction. Right, strategy followed to calculate the interpretability of the model, where the intersection (cyan pixels) represents relevant inferred regions by NaroNet that actually correspond to the synthetic TME containing the disease paradigm.

the sum-to-1 normalized distribution of phenotypes, neighborhoods, and areas in 120 patients, coloured by image field label (Patient type).

| **Optimal parameters found by the architecture search (specific to NaroNet)** | | | |
| --- | --- | --- | --- |
| Number of phenotypes | 10 | Number of neighborhoods | 9 |
| Number of areas | 7 | Global reasoning unit (GLORE) | Activated |
| Patch activation function | True | Max-pooling on patch assignment | Softmax |
| Orthogonal loss | Deactivated | Patch entropy loss | Activated |
| Intra-TME variability function | Deactivated | Patch activation threshold | 0 |
| Patient entropy loss | Deactivated | Mincut entropy loss | Deactivated |
| Lasso loss (last layers) | Deactivated | Graph convolution unit | Resnet |
| Data augmentation (in %) | 0.01% | Number of hops in GNN | 3 |
|  | | | |
| **Optimal parameters found by the architecture search (ML standard parameters)** | | | |
| Batch size | 20 | Weight decay | 0.0001 |
| Learning rate | 0.001 | Dropout rate | 20% |
| Hidden layers | 44 | Normalize patch embeddings (y-axis) | False |
| Normalize patch embeddings (x-axis) | True | Batch normalization | False |

**Supplementary Table 3:** NaroNet optimized hyperparameters after 50 run configurations in endometrial high grade disease paradigm. Since a nested 10-fold cross validation configuration was used for the architecture search and model training and testing, the result of a majority voting of the 10 optimal architectures is shown in the table. Hyperparameter configurations specific to NaroNet are shown above, and below, configuration hyperparameters that are commonly used in most ML-based classifiers.

**Supplementary figure 12**: NaroNet’s prediction performance in a nested 10-fold cross-validation to classify image fields into patient-level labels. The columns (from left to right) represent confusion matrix, ROC curve specifying AUC statistic, and recall-precision curve specifying F1-score and average precision (AP), respectively. **a-c.** Prediction performance to associate image fields with the presence of POLE mutation in two classes, wild-type (WT) and mutated. **d-f.** Prediction performance to associate image fields with 2 tumor histologies, serous-like EC and endometria carcinoma. **g-i.** Prediction performance to associate image fields with MMR deficiency in two classes, stable and unstable. **j-l.** Prediction performance to associate image fields with the copy number variation in two classes, high and low. 10-fold cross-validation using CCI1 disease paradigm experiment. **a,d,g,j.** Confusion matrices comparing patient types. **b,e,h,k.** ROC curves comparing patient types. **c,f,i,l.** Recall precision curves comparing patient types.

**Supplementary figure 13**: Phenotypes learned by NaroNet in endometrial high-grade cancer cohort. **a.** Color legend for each marker. **b.** Heatmap showing the mean marker expression, colored by phenotype **c.** Absolute patch count of each phenotype in the cohort. **d-m.** Left, 12 representative 15x15 pixel patches that were assigned to a specific phenotype, and right, violinplots showing phenotype abundance values across patient types.

**Supplementary figure 14**: Neighborhoods and areas learned by NaroNet related to POLE mutation in endometrial high-grade cancer. **a.** Heatmap showing the Z-scored mean marker expression, colored by neighborhood. **b.** Absolute patch count of each neighborhood in the cohort. **c.** Heatmap showing the Z-scored phenotype composition of neighborhoods. **d.** Heatmap showing the Z-scored neighborhood composition of areas.

**Supplementary figure 15**: Comparison of a cohort-differentiating phenotype found by NaroNet with Qupath quantification. **a.** Correlation of number of cells expressing CD8, PD1, and FoxP3 cells (Qupath) and relative number of patches P2 (NaroNet), each point corresponds to one image. Log-scaled correlation of **a. c.** Boxplot comparing NaroNet’s P2 number of patches in WT and Mutated patient images. **d.** Boxplot comparing Qupath quantification of cells expressing CD8, PD1, and FoxP3 cells in WT and Mutated patient images.

| **Optimal parameters found by the architecture search (specific to NaroNet)** | | | |
| --- | --- | --- | --- |
| Number of phenotypes | 16 | Number of neighborhoods | 24 |
| Number of areas | 17 | Global reasoning unit (GLORE) | Deactivated |
| Patch activation function | False | Max-pooling on patch assignment | Deactivated |
| Orthogonal loss | Deactivated | Patch entropy loss | Activated |
| Intra-TME variability function | Deactivated | Patch activation threshold | 0 |
| Patient entropy loss | Deactivated | Mincut entropy loss | Deactivated |
| Lasso loss (last layers) | Deactivated | Graph convolution unit | Resnet |
| Data augmentation (in %) | 0.1% | Number of hops in GNN | 2 |
|  | | | |
| **Optimal parameters found by the architecture search (ML standard parameters)** | | | |
| Batch size | 18 | Weight decay | 0.0001 |
| Learning rate | 0.01 | Dropout rate | 25% |
| Hidden layers | 44 | Normalize patch embeddings (y-axis) | True |
| Normalize patch embeddings (x-axis) | False | Batch normalization | True |

**Supplementary Table 4:** NaroNet optimized parameters after 50 run configurations in 35-plex mass cytometry images of breast cancer for survival risk assessment. Above, parameter configurations specific of NaroNet, and below, configuration parameters that are commonly used in ML-based classifiers.

**Supplementary Figure 16**: 10-fold nested cross-validation in 35-plex breast cancer risk assessment experiment. **a,d,g.** Confusion matrices for pair combinations of RI, RII, RIII risk classes. **b, e ,h.** ROC curves for pair combinations of RI, RII, RIII risk classes. **c,f,i.** Precision-recall curves for pair combinations of RI, RII, RIII risk classes. **j.** Contrast accuracy curve of PCL module.

**Supplementary Figure 17**: NaroNet on the 35-plex breast cancer assessment experiment. **a.** Heatmap showing the mean marker expression, colored by phenotype. **b,c,d.** Relative abundance of N4, P2, and P5 in RI-RIII survival risk groups.
